# The photoregulation of a mechanochemical polymer scission

**DOI:** 10.1038/s41467-018-05996-7

**Published:** 2018-08-29

**Authors:** Jumpei Kida, Keiichi Imato, Raita Goseki, Daisuke Aoki, Masakazu Morimoto, Hideyuki Otsuka

**Affiliations:** 1 Department of Chemical Science and Engineering, Tokyo Institute of Technology, 2-12-1 Ookayama, Meguro-ku, Tokyo 152-8550 Japan; 2 Department of Chemistry, Rikkyo University, 3-34-1 Nishi-Ikebukuro, Toshima-ku, Tokyo 171-8501 Japan

## Abstract

Control over mechanochemical polymer scission by another external stimulus may offer an avenue to further advance the fields of polymer chemistry, mechanochemistry, and materials science. Herein, we demonstrate that light can regulate the mechanochemical behavior of a diarylethene-conjugated Diels–Alder adduct (DAE/DA) that reversibly isomerizes from a weaker open form to a stronger closed form under photoirradiation. Pulsed ultrasonication experiments, spectroscopic analyses, and density functional theory calculations support the successful photoregulation of the reactivity of this DAE/DA mechanophore, which is incorporated at the mid-chain of a polymer, and indicate that higher force and energy are required to cleave the closed form of the DAE/DA mechanophore relative to the open form. The present photoregulation concept provides an attractive approach toward the generation of new mechanofunctional polymers.

## Abstract (web summary)

Control over mechanochemical polymer scission by external stimuli may offer an avenue to further advance the fields of polymer chemistry, mechanochemistry, and materials science. Here the authors show light regulating the mechanochemical behavior of a diarylethene-conjugated Diels–Alder adduct.

## Introduction

Mechanophores, i.e., force–responsive molecules, represent a defining feature of the remarkable advancements that have been accomplished in polymer research in the past decade.^1,2^ Embedding mechanophores into polymer backbones enables the transfer of an applied force through them, providing a new powerful platform to endow polymeric materials with fascinating functionalities, such as chromism,^3–7^ luminescence,^8–12^ strengthening by cross-linking,^7,13–15^ activation of latent metal catalysts,^13,14,16–20^ and the generation of reactive species^21,22^ in response to force instead of heat or light. The focus of previous mechanophore studies can be broadly categorized into the search for novel mechanophores and the development of unique functionalities originated from them in polymers^2,23–26^ or the elucidation of the influence of the mechanophore structure, its peripheral environment, and macromolecular structure on the mechanophore activation.^27–37^ This innovative research has significantly advanced the emerging field of polymer mechanochemistry. To the best of our knowledge, successful examples of the regulation of mechanochemical polymer reactions by another external stimulus have not been reported so far.

**Fig. 1:**
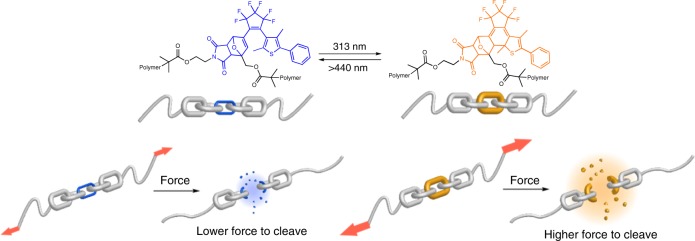
Conceptual illustrations. Photoregulated mechanochemical polymer scission based on DAE/DA

Here, we report an example for the photoregulation of the mechanochemical reactivity of a mechanophore. In the present study, we focused on a Diels–Alder (DA) adduct, which can be regarded as a representative group of mechanophores,^37–41^ and regulated the mechanical cleavage of the DA bond by another external stimulus. DA adducts that emit fluorescence or release small molecules upon mechanical cleavage of the DA bond have already been developed, and these have provided deep insight into the fundamental aspects of polymer mechanochemistry.^11,37,42,43^ We envisaged that control over the mechanical cleavage could possibly be established by photoisomerization of a photochromic diarylethene (DAE).^44^ Recently, control over the thermally induced bond cleavage of DAE-conjugated DA adducts (DAE/DA) has been reported using a light stimulus,^45–50^ and this approach also holds promise for mechanically induced retro-DA reactions. The structure of the DAE/DA unit used in this study is shown in Fig. 1. We expected that the open form (DAE-open/DA), when incorporated in a polymer chain, would experience mechanical scission via a retro-DA reaction upon sonication of its solution; an event that would be less likely for the closed isomer (DAE-closed/DA). Therefore, we synthesized a polymer, which contained a DAE/DA moiety at the mid-chain, and investigated the chain scission by sonication before and after irradiation with UV light. The difference in the scission rates of the polymers bearing the open and closed isomers demonstrates the potential of this system to regulate other properties that involve the application of force.

## Results

### Synthesis and model photoregulation reaction of (DAE-open/DA)-diol

**Fig. 2:**
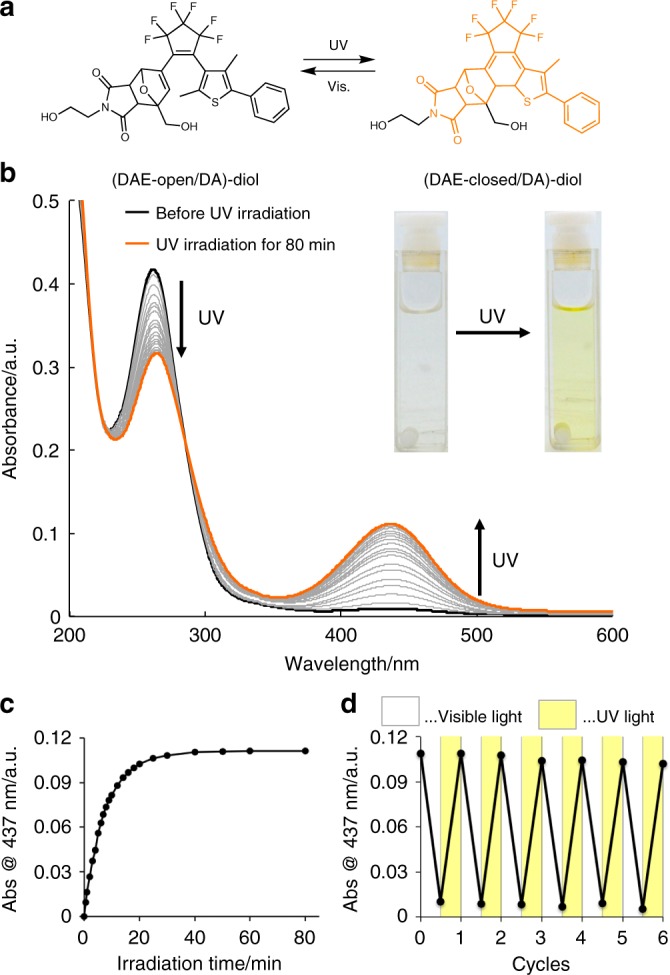
Model photoregulation reaction of (DAE-open/DA)-diol. **a** Reversible photoisomerization of DAE/DA-diol. **b** Time dependence of the UV–vis absorption spectra of an acetonitrile solution of DAE/DA-diol (22.7 µmol L^–1^) under irradiation with UV light (λ = 313 nm). **c** Time dependence of the absorption at 437 nm under irradiation with UV light (λ = 313 nm). **d** Absorption changes at 437 nm under alternating irradiation with UV (313 nm; 1 h) and visible light (> 440 nm; 10 min)

(DAE-open/DA)-diol, i.e., a model DAE/DA unit and the polymer precursor, was synthesized in five steps (see Fig. 2a for structure, Supplementary Method for synthesis, Supplementary Figs. 19–33 for NMR spectra, and Supplementary Fig. 1 for thermal reactivity). The photoisomerization of this unit was investigated by UV–vis absorption measurements, prior to “locking” and “unlocking” its retro-DA reaction by sonication after insertion in a polymer chain. Upon exposure to UV light (λ = 313 nm), the intensity of the absorption band with a maximum at 260 nm, which corresponds to (DAE-open/DA)-diol, gradually decreased, while a new broad band emerged with a maximum at 437 nm (Fig. 2b). Extension of the π-conjugation in the DAE segment by isomerization from the open to the planar closed form causes such a bathochromic shift of the absorption, which is consistent with the observed color change (colorless→yellow) of the solution (inset in Fig. 2b). The ring-closing reaction reached a photostationary state (PSS) after 60 min of irradiation with UV light (Fig. 2c); the PSS corresponds to ca. 50% of (DAE-closed/DA)-diol, as determined by comparing the integral area of the signals in the ^1^H NMR spectrum (Supplementary Fig. 2). Almost all closed molecules in the mixture returned to the open form after 10 min of irradiation with visible light (λ > 440 nm) (Supplementary Fig. 3); this reversible isomerization was observed repeatedly by monitoring the absorbance at 437 nm (Fig. 2d). These results suggest the potential to reversibly lock and unlock the mechanochemical retro-DA reaction in the DAE/DA unit.

### Synthesis of polymers

**Fig. 3:**
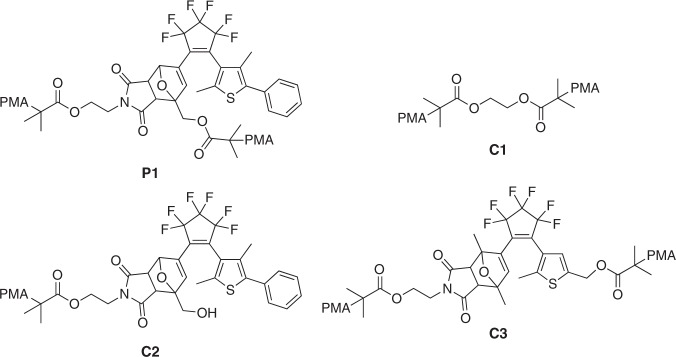
Chemical diagrams. Polymer structures used in this study

Since the model reaction using (DAE-open/DA)-diol proceeded successfully, a series of polymers containing the DAE/DA unit were synthesized. A linear polymer with a DAE/DA functionality at the mid-chain (**P1**) was synthesized via esterification of (DAE-open/DA)-diol with 2-bromoisobutyryl bromide, followed by living radical polymerization of methyl acrylate (MA) from the two initiating sites (see Fig. 3 for the structure and Supplementary Fig. 4 for thermal reactivity). Three linear PMAs were prepared as control samples: one without DAE/DA (**C1**), one containing a DAE/DA unit located at a chain end (**C2**) (Supplementary Fig. 5), and one where the polymer chains are attached across the DAE/DA unit (**C3**) (Supplementary Fig. 6), whereas the PMA chains in **P1** are linked only to the DA part. **C1** should enable the estimation of the relative mechanochemical reactivity of both isomers, **C2** should preclude any substantial transferal of force into the DAE/DA unit and eliminate any concerns regarding localized heating, and **C3** will help to understand the mechanisms of both the DA and DAE mechanochemistry. All the polymers used in this study exhibited a number-average molecular weight (*M*_n_) of 131–149 kDa with polydispersity indices (PDI) of 1.17–1.23 (Supplementary Table 1).

### Sonication of the polymers

**Fig. 4:**
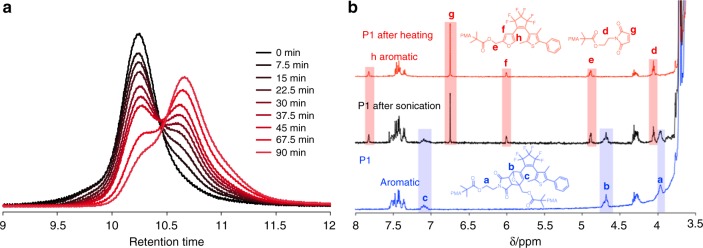
Mechanical retro-DA reaction of **P1**. **a** SEC curves for **P1** upon sonication. A 2 mg mL^–1^ polymer solution was exposed to pulsed ultrasound (10.6 W cm^–2^) under an atmosphere of argon in an ice bath. **b** ^1^H NMR spectra of **P1** before and after sonication (45 min), as well as after heating (3 h), showing the products of the retro-DA reaction (Supplementary Fig. 4)

The mechanochemical reactivity of these polymers in acetonitrile was evaluated using pulsed ultrasonication (10.6 W cm^–2^) in a water ice bath. **P1** underwent a steady decrease in molecular weight with increasing sonication time, which was monitored by size-exclusion chromatography (SEC) (Fig. 4a). The initial polymer peak (*M*_n_ = 136 kDa) was attenuated upon sonication, while a new well-defined peak appeared at approximately half of the original molecular weight (*M*_n_ = 68 kDa). Mechanochemical scission of **P1** was further investigated by ^1^H NMR spectroscopy. Upon sonication for 45 min, the ^1^H NMR spectrum of **P1** showed new signals at 4.12, 4.95, 6.07, 6.81, and 7.89 ppm (Fig. 4b), which is consistent with the signals of the retro-DA products, i.e., furan and maleimide groups, that were also observed in a heat-induced retro-DA reaction. This result clearly indicates that sonication of **P1** induces a retro-DA reaction in the mid-chain DAE/DA unit. A rough comparison of the two peak areas in the SEC curve revealed that 50% of the polymer chains were cleaved after sonication for 45 min. As estimated from the integrals in the ^1^H NMR spectrum, 80% corresponded to a selective cleavage at the DAE/DA unit through the retro-DA reaction, while 20% should be attributed to random cleavages in the PMA backbone. In contrast, no new peaks resulting from a retro-DA reaction were observed after sonication of **C2** (bearing an end-chain DAE/DA unit) as well as of **C1** (without a DAE/DA functionality) (Supplementary Figs. 10, 11). These results clearly confirm that the applied mechanical force is responsible for the selective scission at the DAE/DA unit in **P1**, thus precluding other alternative pathways such as thermal activation. Surprisingly, sonication of **C3**, where the PMA chain is attached to both the DAE and DA fragments, did not afford selective cleavage through a retro-DA reaction (Supplementary Fig. 12). To investigate the reasons behind the mechanochemical inertness of the DAE/DA unit in **C3**, we performed CoGEF calculations on the DAE/DA models (**MP1** and **MC3**) incorporated in **P1** and **C3**, as well as on the **C1** PMA chain (**MC1**). The cleavage in **MC3** required much higher energy and force (*E*_max_ = 770 kJ mol^–1^ and *F*_max_ = 5.19 nN) than those for **MP1** (236 kJ mol^–1^/3.88 nN) (Supplementary Figs. 16, 17, and Supplementary Table 2). The energy and force required for the cleavage of the **C1** PMA chain (647 kJ mol^–1^/5.15 nN) were higher than those for **P1** but lower than those for **C3** (Supplementary Fig. 15). These results indicate that preferential cleavage occurs at the DAE/DA unit prior to random PMA cleavage under sonication in the case of **P1**, while sonication of **C3** affords random cleavage as the mid-chain DAE/DA unit is extremely inert toward elongational forces. These findings are consistent with reports by Bo et al.^37^ and Makarov et al.^51^, who showed that cleavage of DA isomers with distal geometry (similar to that of the unit in **C3**) requires higher energy and force than cleavage in the case of proximal geometry (similar to that of **P1**).

### Photoregulation of the mechanical polymer scission

**Fig. 5:**
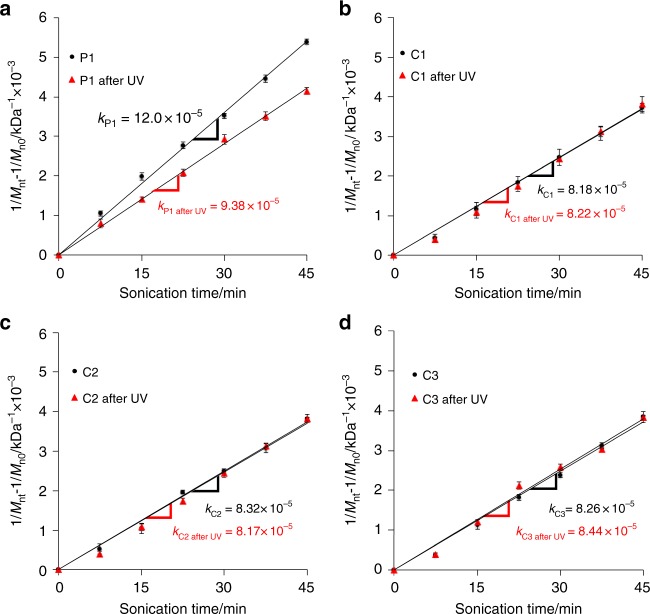
Rate of polymer chain scission upon exposure to pulsed ultrasound. **a–d** Cleavage rate of **P1**, **C1**, **C2**, and **C3** before and after irradiation with UV light (313 nm, 1 h). A 2 mg mL^–1^ polymer solution was sonicated with pulsed ultrasound (10.6 W cm^–2^) under an atmosphere of argon in an ice water bath. Measurements were performed in triplicate, and the error bars represent a standard deviation

To demonstrate the photoregulation of the mechanochemical cleavage of the DA bond by DAE isomerization at the chain center, we determined the rate constants for the chain scission in the sonicated polymer solutions before and after irradiation with UV light using SEC measurements (see Supplementry Information Equation (2) for detail). The cleavage rate constant for **P1**, i.e., the slope of the black line in Fig. 5a, was 12.0 × 10^–5^ min^–1^ kDa^–1^; exposure to UV light (λ = 313 nm) for 1 h to form **P1-closed** decreased the rate constant to 9.38 × 10^–5^ min^–1^ kDa^–1^ (see SI for details). After UV irradiation, **P1** contained 60% of ring-closed DAE/DA, as calculated from the UV–vis spectrum of the solution using the molar absorptivity of (DAE-closed/DA)-diol at 437 nm (Supplementary Fig. 7), and the individual cleavage rate constant of **P1-closed** was accordingly estimated to be 7.63 × 10^–5^ min^–1^ kDa^–1^. This rate is lower than that of **C1** (8.18 × 10^–5^ min^–1^ kDa^–1^), which barely changed after UV irradiation (Fig. 5b). In the UV–vis measurements of **P1** after UV irradiation under sonication, an attenuation of the absorption at 437 nm was observed, which was attributed to the presence of DAE-closed/DA (Supplementary Fig. 13), indicating the slow destruction of the DAE-closed/DA skeleton. In contrast to **P1**, the rate constant of **C2** was almost that of **C1** and barely decreased upon exposure to UV light (Fig. 5c), while 43% of the DAE/DA unit at the chain end isomerized into the closed form (Supplementary Fig. 8). Moreover, the UV–vis spectrum of **C2** after exposure to UV light remained unchanged upon sonication (Supplementary Fig. 14a). These results indicate that random cleavage occurs except at the chain-end DAE/DA isomers, and that the DAE/DA unit in **P1** is responsible for the successful photoregulation of the mechanical chain scission. Despite the mid-chain functionality of **C3**, the rate constant corresponds to the random cleavage of PMA, which remained almost unchanged after exposure to UV light accounting for 53% of the isomerization (Supplementary Fig. 9) of the DAE/DA unit (Fig. 5d). A destruction of the closed skeleton under sonication was not observed in the UV–vis measurements of **C3** (Supplementary Fig. 14b). These results indicate that the closed DAE isomer is inert toward elongational mechanical forces, although other DAE units that are bound differently to a polymer chain may serve as mechanophores.

To demonstrate the reversibility of the photoregulated mechanochemical scission rate, we alternately irradiated a **P1** solution with UV (λ = 313 nm) and visible (λ > 440 nm) light during intermittent sonication. The scission rate declined upon exposure to UV light but recovered after irradiation with visible light (Supplementary Fig. 18). This procedure was repeated for several cycles, although the rate for **P1** scission slightly decreased with each cycle. This is probably due to the generation of some non-scission byproducts via the destruction of the DAE-close/DA unit upon sonication of the UV-irradiated **P1** solution. Nevertheless, it has been clearly demonstrated that the mechanoreactivity of the DAE/DA mechanophore can be reversibly changed using light as a key.

## Discussion

The development of a mechanophore having the feature enabling control its mechanical responsiveness with external stimuli requires accurate and flexible designing of mechanophore. The fact that the thermal retro-DA reaction of DAE/DA-diol was controllable with irradiation of light suggests that control of mechanical retro-DA reaction is also possible. The progress of retro-DA reaction with the ultrasonication for polymer containing DAE/DA at the mid-chain (**P1**) verified that DAE/DA works as a mechanophore. The photo-irradiation for **P1** induced the decreasing of cleavage rate of polymer chain. This change derives from the ring-closing reaction of DAE inducing strengthen of structure of DAE/DA mechanophore. The experiment using DAE/DA having different polymer connecting point (**C3**) (not work as mechanophore) remarkably prove the change in cleavage rate of **P1** derived from not the change in steric structure of DAE, but the specific character of DAE/DA. The property controlling strength of chemical bond to mechanical stress will enable us to create the innovative material that can change the mechanical property with light. The results presented in this paper enable others to quest for such materials in parallel with our own ongoing effort.

In summary, combining a well-known DA mechanophore with a photochromic DAE unit has provided a photoregulated lock for its mechanoreactivity (retro-DA reaction). The scission rate of the polymer bearing a DAE/DA moiety at the mid-chain was reversibly regulated by sonication using irradiation with UV and visible light. This is the first example of how to control mechanophore reactivity using another external stimulus. Since a wide variety of polymer systems containing DA mechanophores have been developed, we are convinced that our mechanoreactivity lock has the potential to be a common tool for the regulation of the fascinating force-utilizing properties of such systems.

## Methods

### Preparation of polymers containing DAE/DA

The synthesis, purification, and characterization, which includes the NMR, IR, and GPC data, mechanical characterization, control experiments, and additional details for all polymers, are described in detail in Supplementary Information.

### Photochemical reactions

The photochemical reactions were performed employing a Xe lamp equipped with either an optical filter at 313 nm (ring-closing reactions) or an optical glass > 440 nm (ring-opening reactions). The reactions were carried out in anhydrous acetonitrile at concentrations below 30 µmol L^–1^.

### Sonication

Ultrasound experiments were performed in anhydrous acetonitrile using a Branson sonifire model 250D with a 1.1 cm diameter titanium probe on solutions of the polymers at a concentration of 2 mg mL^–1^. The solutions were degassed by argon sparging (30 min) prior to sonication and the argon atmosphere was retained during the experiments. Pulsed ultrasound was delivered at a power of 10.6 W cm^–2^ with on/off periods of one second.

## Electronic supplementary material

Supplementary Information

## Data Availability

All data supporting the findings of this study are available within the article and its Supplementary Information. All other data are available from the corresponding author upon reasonable request.
