# Supplementary material for: The photoregulation of a mechanochemical polymer scission
Source: Nat Commun. 2018 Aug 29;9:3504. doi: 10.1038/s41467-018-05996-7 (PMC6115466; doi:10.1038/s41467-018-05996-7)
Supplement: Supplementary file 1 — Supplementary Information [file 41467_2018_5996_MOESM1_ESM.pdf]

Supplementary Information for

The photoregulation of a mechanochemical polymer scission

by Kida et al.

Supplementary Methods

General Experimental Details

All solvents and reagents were purchased from Sigma-Aldrich, Wako Pure Chemical Industries, Tokyo Chemical Industry, or Kanto Chemical and used as received, unless otherwise noted. Methyl acrylate was purified by basic aluminum chromatography to remove the polymerization inhibitor. Cu(0) wire (diameter: 1.0 mm, purity >99.9%) was washed with H_2SO_4 just before use to purify the surface.

Ultrasound experiments were performed using a Branson sonifire model 250D with a 1.1 cm diameter solid probe. The distance between the titanium tip and bottom of the glass cell was 1 cm. The ultrasonic intensity was calibrated using the method outlined by Hickenboth *et al.*[1]

^{1}H NMR spectra were measured at 25 °C using a 300 MHz Bruker spectrometer or 500 MHz Bruker spectrometer with tetramethylsilane (TMS) as an internal standard in chloroform-*d* ($CDCl_3$), DMSO-d_6 or acetonitrile-d_3 (CD_3CN). IR spectra were obtained with a Perkin-Elmer Spectrum One infrared spectrometer. Size exclusion chromatography (SEC) measurements were carried out at 40 °C on TOSOH HLC-8320 SEC system equipped with a guard column (TOSOH TSK guard column Super H-L), three columns (TOSOH TSK gel SuperH 6000, 4000, and 2500), a differential refractive index detector, and a UV-vis detector. Tetrahydrofuran (THF) was used as the eluent at a flow rate of 0.6 mL/min. Polystyrene (PS) standards (M_n = 4430−3242000; M_w/M_n = 1.03−1.08) were used to calibrate the SEC system. UV/vis absorption spectroscopy was performed on a JASCO V-650 spectrophotometer at 25 °C. Photochemical reactions were carried out employing a USHIO UXL-500SX Xe lamp equipped with either ASAHI SPECTRA optical filter at 313 nm for ring-closing and Hoya colored optical glass Y44 for ring-opening reactions.

Reactivity of DAE/DA

Retro-Diels–Alder reaction of (DAE-open/DA)-diol

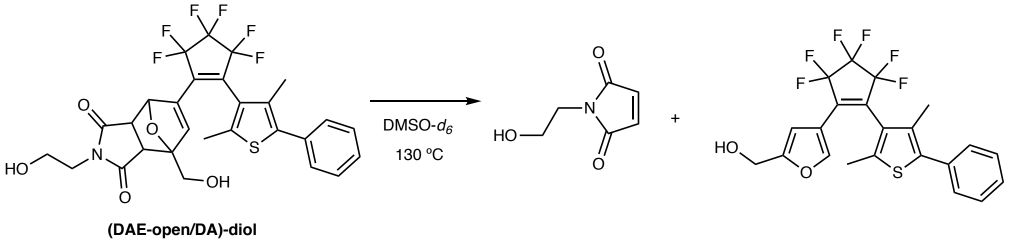

DMSO-d_6 (1.00 mL) solution of **(DAE-open/DA-diol**) (5.00 mg, 8.34 μmol) was stirred at 130 ºC for 1 h. The reaction progress was monitored by ^{1}H NMR measurement (Supplementary Fig. 1). ^{1}H NMR showed quantitative progress of retro-DA reaction.

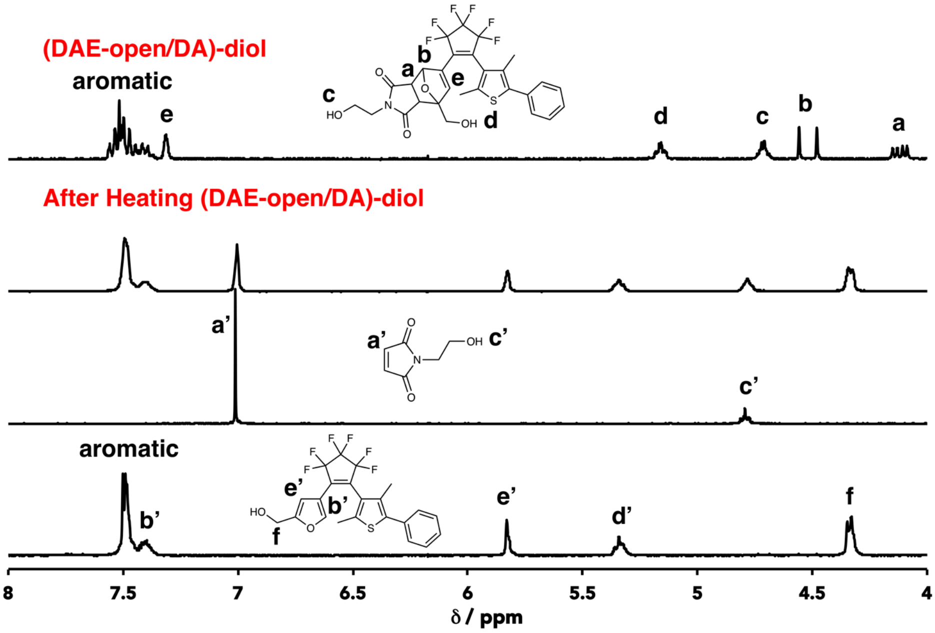

Supplementary Fig. 1. ^{1}H NMR spectra of **(DAE-open/DA)-diol** before and after heating at 130 ºC for 1 h and along with reference compound (300 MHz, DMSO-d_6, 25 ºC).

Ring-closing reaction of DAE/DA-diol

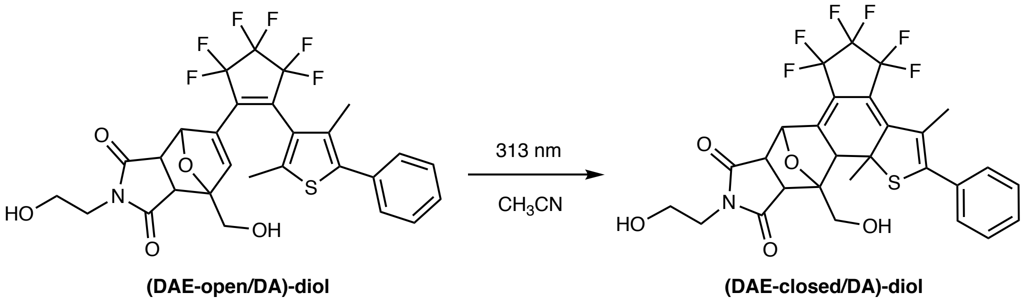

Acetonitrile (300 mL) solution of (**DAE-open/DA)-diol** (5.00 mg) was exposed to UV light at 313 nm for 1h.

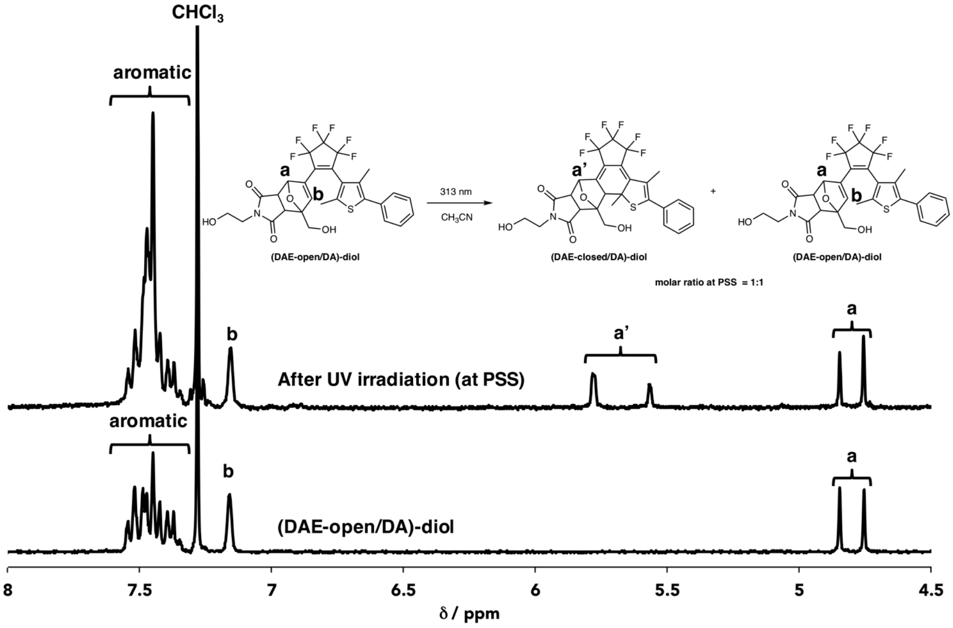

Supplementary Fig. 2. ^{1}H NMR spectra (300 MHz, $CDCl_3$, 25 ºC) of model photo isomerization in **(DAE-open/DA-diol)** to form ring-closed isomer with UV light irradiation. The mixture at photo stationary state (PSS) contained ca. 50% of ring-closed isomer, estimated from comparison between integration value of signals **a'** and **a**.

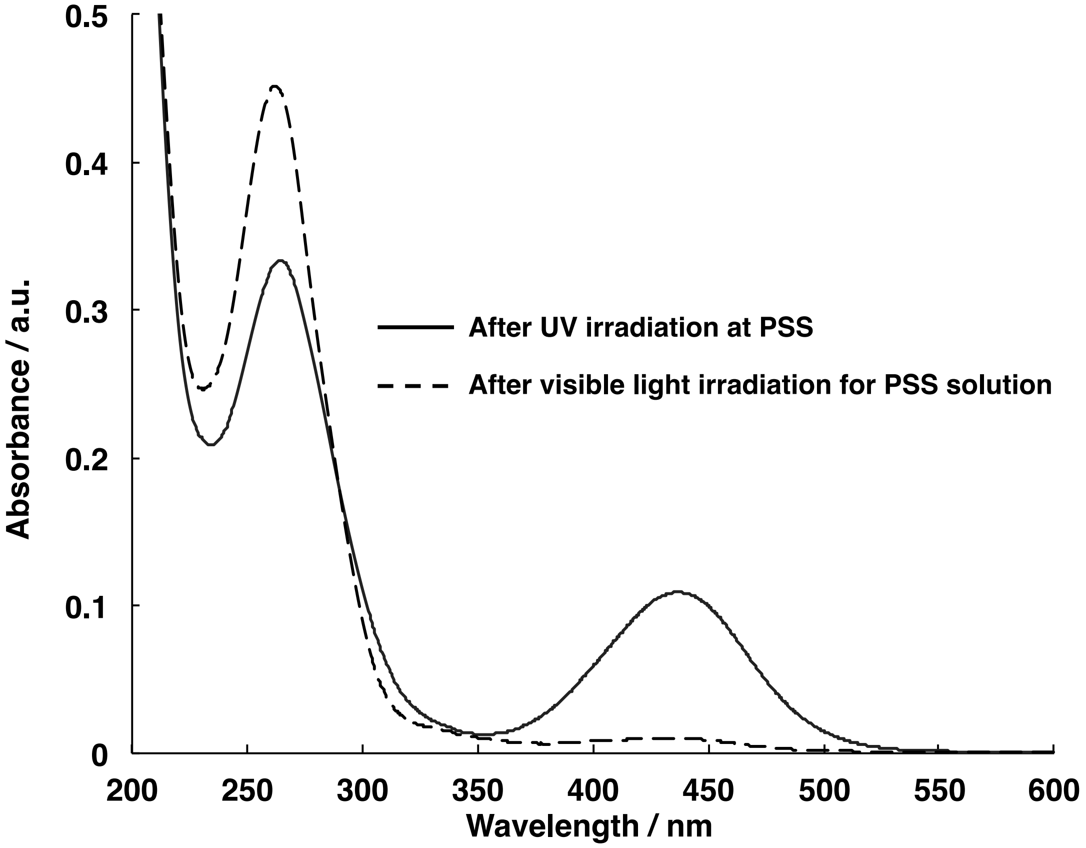

Supplementary Fig. 3. UV-Vis spectra of **DAE/DA-diol** at PSS (solid line) and after visible light irradiation for 10 min for PSS solution (dashed line).

General procedure for the synthesis of poly(methyl acrylate) (PMA) containing a chain-centerd initiating unit.

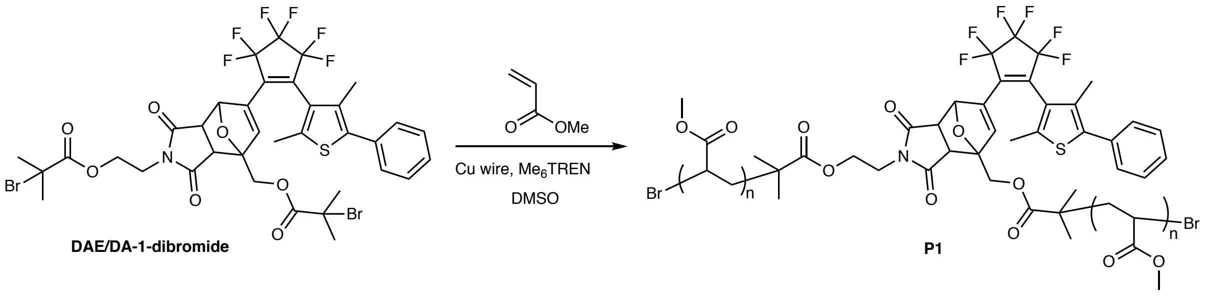

A representative procedure for the synthesis of polymer **P1** is provided. A 30 mL Schlenk flask equipped with a stir bar was charged with freshly cut copper wire (0.75 cm×2 length, 1.0 mm in diameter) and initiator **DAE/DA-dibromide** (13.3 mg, 14.8 μmol). The flask was sealed with a stopcock followed by the addition of DMSO (6.2 mL) and methyl acrylate (3.1 mL, 334 mmol) via syringe. The solution was deoxygenated via four freeze-pump-thaw cycles and after the final cycle, the flask was warmed to room temperature and backfilled with nitrogen. Me_6TREN (16.0 μL, 59.2 μmol) was added using a micro syringe to initiate the polymerization. After stirring at room temperature for 140 minutes, the flask was opened to air and the solution was diluted with chloroform. Dissolved Cu catalyst was removed with neutral alumina column chromatography. The polymer solution was precipitated into cold methanol and the isolated material was dried thoroughly under vacuum to provide 1.40 g of polymer **P1** (47%).

The synthesis of poly(methyl acrylate) (PMA) containing the DAE/DA unit at the chain-end (C2).

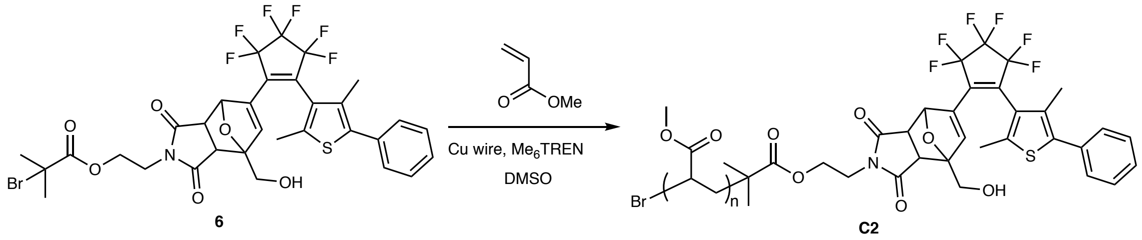

A 30 mL Schlenk flask equipped with a stir bar was charged with freshly cut copper wire (0.75 cm×2 length, 1.0 mm in diameter) and initiator **6** (11.2 mg, 15.0 μmol). The flask was sealed with a stopcock followed by the addition of DMSO (6.2 mL) and methyl acrylate (3.1 mL, 334 mmol) via syringe. The solution was deoxygenated via four freeze-pump-thaw cycles and after the final cycle, the flask was warmed to room temperature and backfilled with nitrogen. Me_6TREN (16.0 μL, 59.2 μmol) was added using a micro syringe to initiate the polymerization. After stirring at room temperature for 140 minutes, the flask was opened to air and the solution was diluted with chloroform. Dissolved Cu catalyst was removed with neutral alumina column chromatography. The polymer solution was precipitated into cold methanol and the isolated material was dried thoroughly under vacuum to provide 1.51 g of **C2** (50%).

Supplementary Table 1. List of polymers synthesized

Polymer	M_n/kDa	PDI
P1	136	1.20
C1	149	1.17
C2	131	1.23
C3	141	1.23

Thermal retro-DA reaction of P1

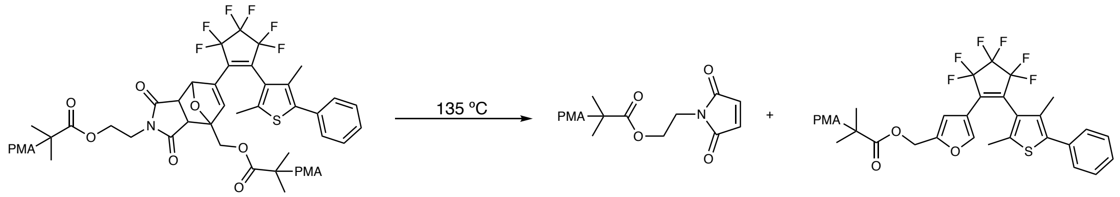

P1 (50.0 mg, M_n =136 kDa) was heated at 135 °C for 3 h. The reaction progress was monitored by ^{1}H NMR and SEC measurements (Supplementary Fig. 4). The ^{1}H NMR spectrum showed the progress of retro-DA reaction and SEC curve shifted to lower molecular weight region, and the estimated molecular weight was half of the original, which indicated that DAE/DA unit is at the center of polymer chain.

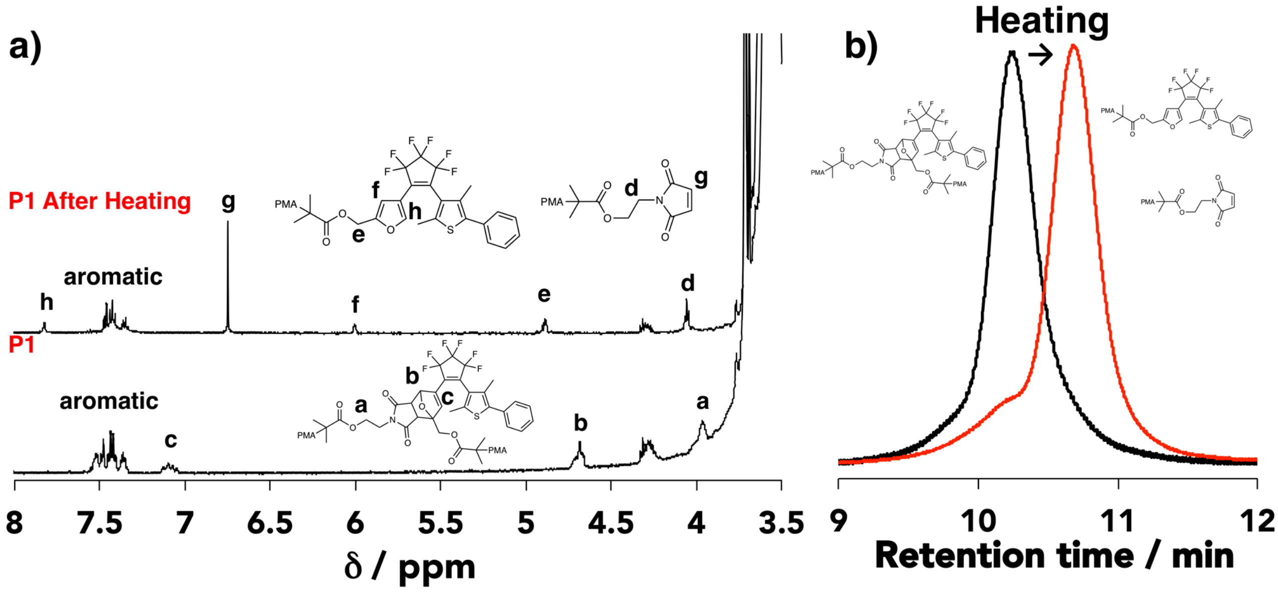

Supplementary Fig. 4. a) ^{1}H NMR spectra (500 MHz, CD_3CN, 25 °C) of **P1** before (lower) and after (upper) heating 135 °C for 3 h and b) SEC curves of **P1** before (black) and after (red) heating 135 °C for 3h.

Thermal retro-DA reaction of C2

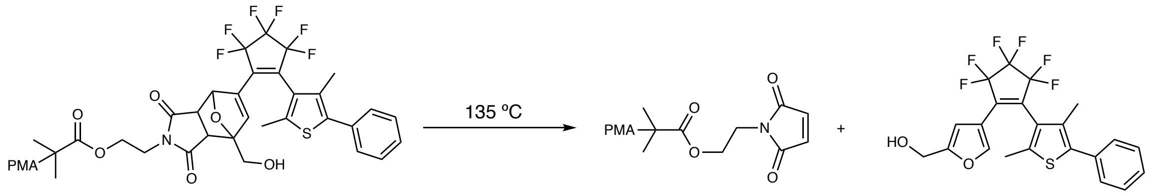

C2 (50.0 mg, M_n = 131 kDa) was heated at 135 °C for 3 h. The reaction progress was monitored by ^{1}H NMR and SEC measurements (Supplementary Fig. 5). ^{1}H NMR spectra showed the progress of retro-DA reaction. SEC curves did not change before and after reaction, which indicated that DAE/DA unit is at the polymer chain end.

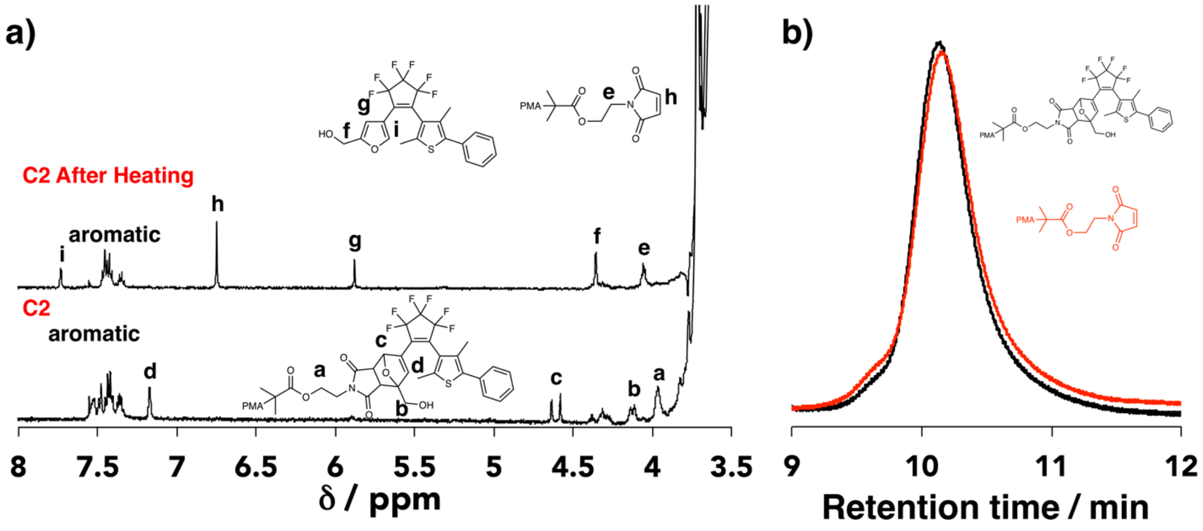

Supplementary Fig. 5. a) ^{1}H NMR spectra (500 MHz, CD_3CN, 25 ºC) of **C2** before (lower) and after (upper) heating 135 ºC for 3 h and b) SEC curves of **C2** before (black) and after (red) heating 135 ºC for 3h.

Thermal retro-DA reaction of C3

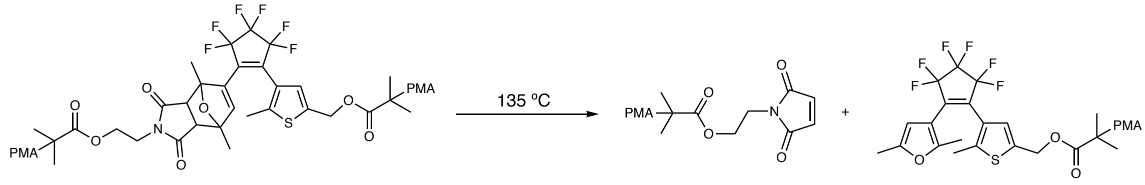

C3 (50.0 mg, M_n = 141 kDa) was heated at 135 ºC for 3 h. The reaction progress was monitored by ^{1}H NMR and SEC measurements (Supplementary Fig. 6). ^{1}H NMR spectra showed the progress of retro-DA reaction and SEC curves shifted to lower molecular weight region, and the estimated molecular weight was half of the original, which indicated that DAE/DA unit is at the center of polymer chain.

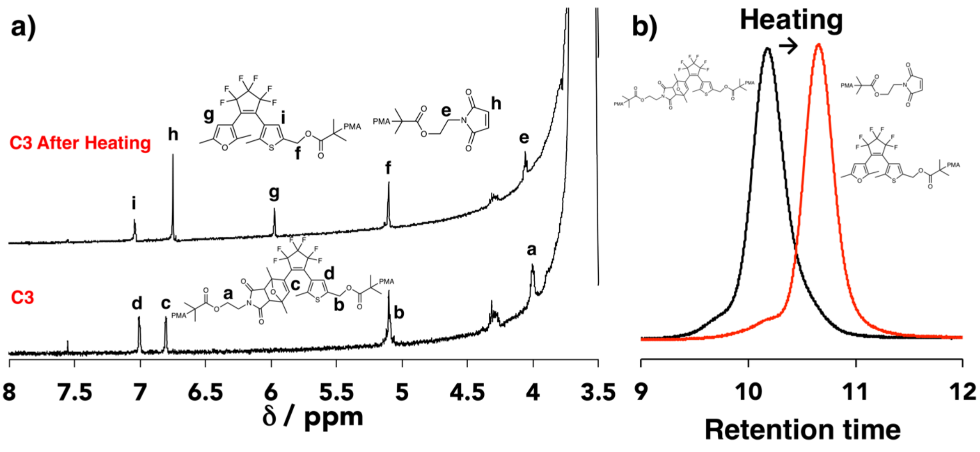

Supplementary Fig. 6. a) ^{1}H NMR spectra (500 MHz, CD_3CN, 25 ºC) of **C3** before (lower) and after (upper) heating 135 ºC for 3 h and b) SEC curves of **C3** before (black) and after (red) heating 135 ºC for 3h.

Photoisomerization of P1

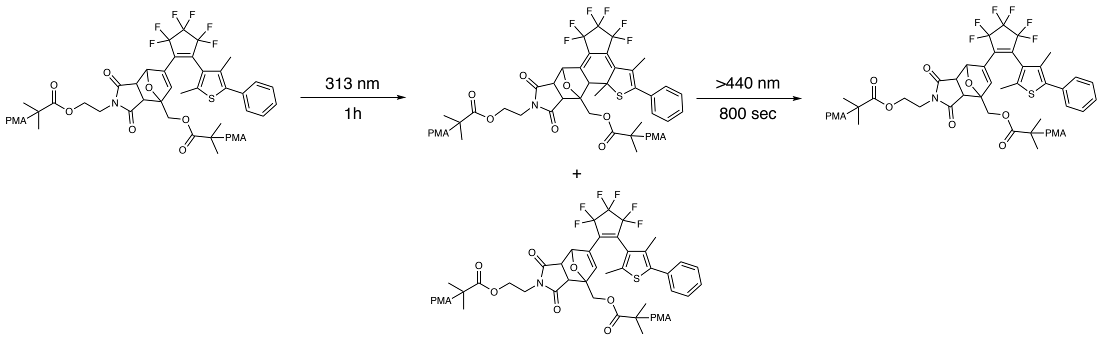

An acetonitrile (5 mL) solution of **P1** (10.0 mg, M_n = 136 kDa) was exposed to UV light at 313 nm for 1 h. Subsequently, the resulting mixture was irradiated with visible light at >440 nm for 800 sec. These reaction progresses were monitored by UV-Vis measurement. **P1** after UV irradiation contained 60% of the ring-closed DAE/DA, which was calculated from the UV-Vis spectrum of the solution using the molar absorptivity of (DAE-closed/DA)-diol (ε = 480 m^2/mol, calculated by Lambert–Beer equation (Supplementary Equation 1), where *Abs* is the absorption at 437 nm, ε is molar absorptivity (dm^2/mol), c is concentration (mol/L), and l is the optical path length of quartz cell).

$$Abs = \varepsilon c l \qquad (1)$$

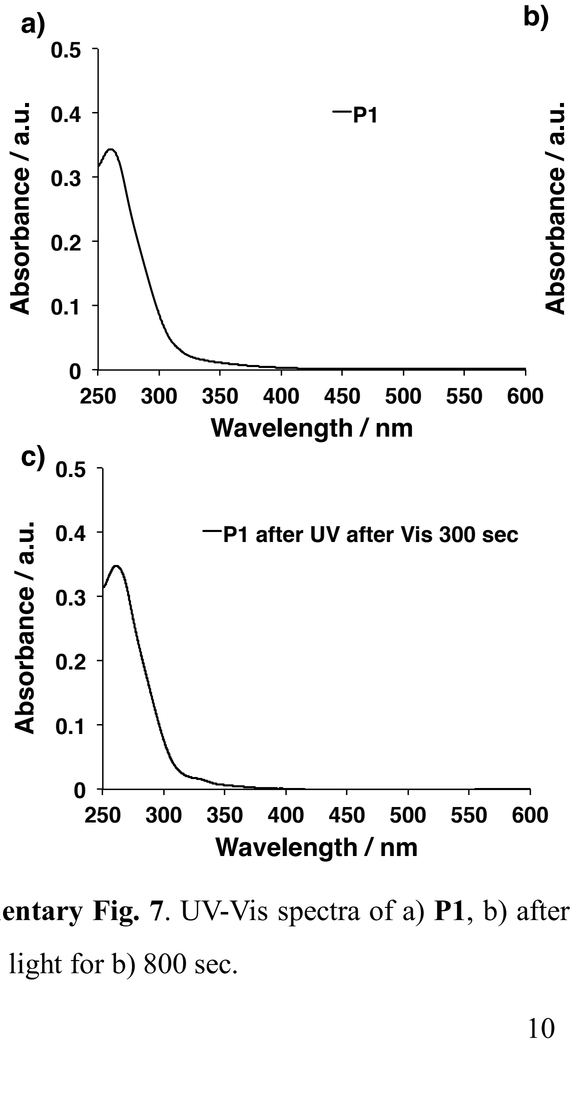

Supplementary Fig. 7. UV-Vis spectra of a) **P1**, b) after UV irradiation of a) for 1 h, and c) after irradiation of visible light for b) 800 sec.

Photoisomerization of C2

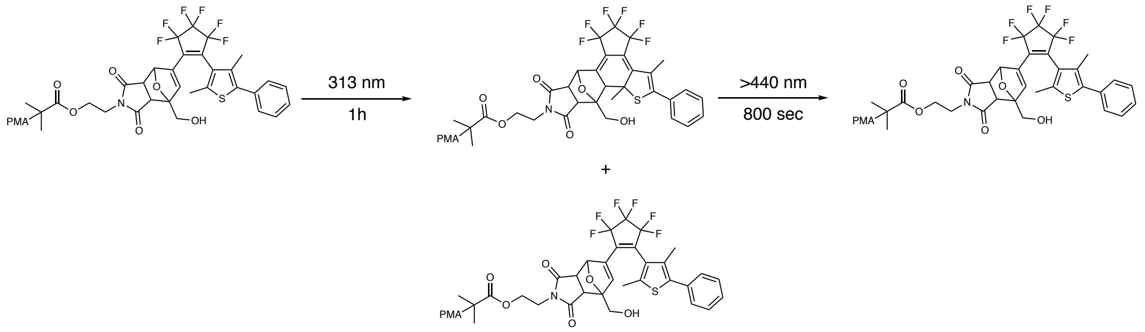

An acetonitrile (5 mL) solution of **C2** (10.0 mg, M_n = 131 kDa) was exposed to UV light at 313 nm for 1 h. Subsequently, the resulting mixture was irradiated with visible light at >440 nm for 800 sec. These reaction progresses were monitored by UV-Vis measurement. **C2** after UV irradiation contained 43% of the ring-closed DAE/DA, which was calculated from the UV-Vis spectrum of the solution using the molar absorptivity of (DAE-closed/DA)-diol (ε = 480 m^2/mol, calculated by Supplementary Equation 1).

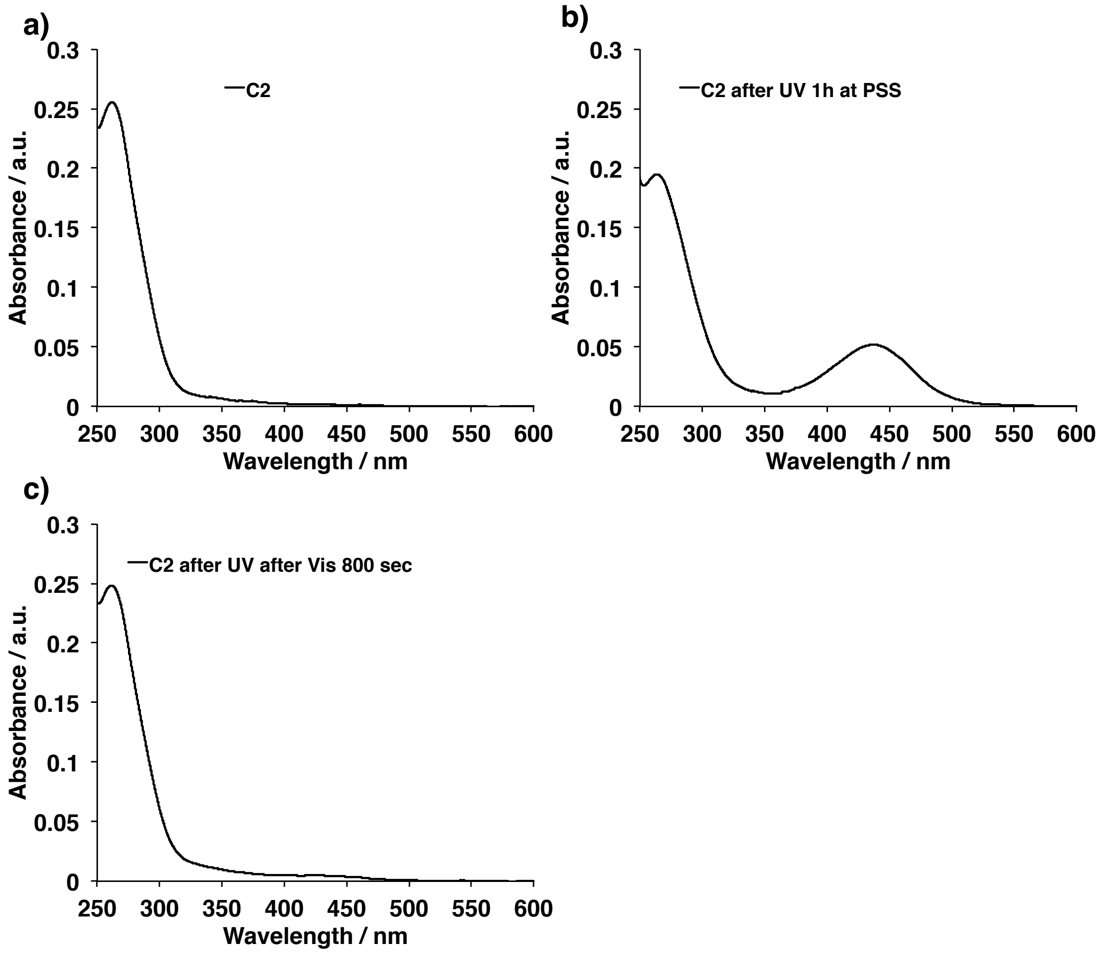

Supplementary Fig. 8. UV-Vis spectra of a) **C2**, b) after UV irradiation of a) for 1 h, and c) after irradiation of visible light for b) 800 sec.

Photoisomerization of C3

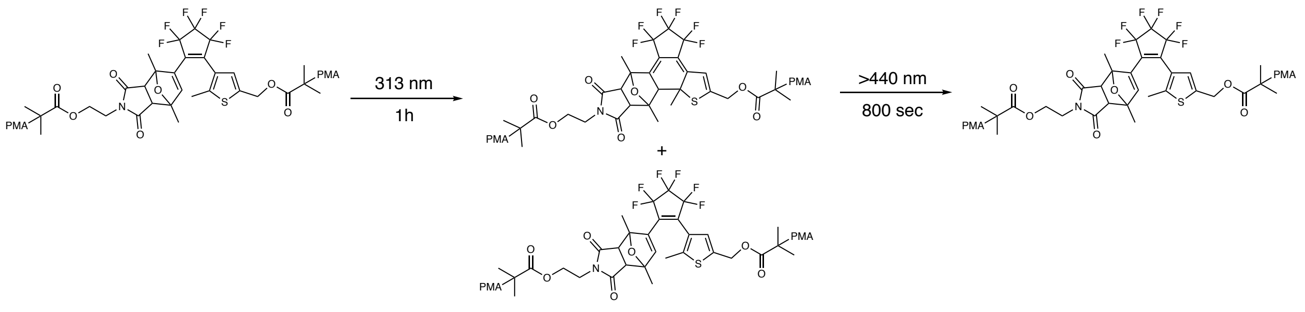

An acetonitrile (5 mL) solution of **C3** (10.0 mg, M_n = 141 kDa) was exposed to UV light at 313 nm for 1 h. Subsequently, the resulting mixture was irradiated with visible light at >440 nm for 800 sec. These reaction progresses were monitored by UV-Vis measurement. **C3** after UV irradiation contained 53% of the ring-closed DAE/DA, which was calculated from the UV-Vis spectrum of the solution using the molar absorptivity of DAE/DA-diol-2 [m] (ε = 467 m^2/mol, calculated by Supplementary Equation 1).

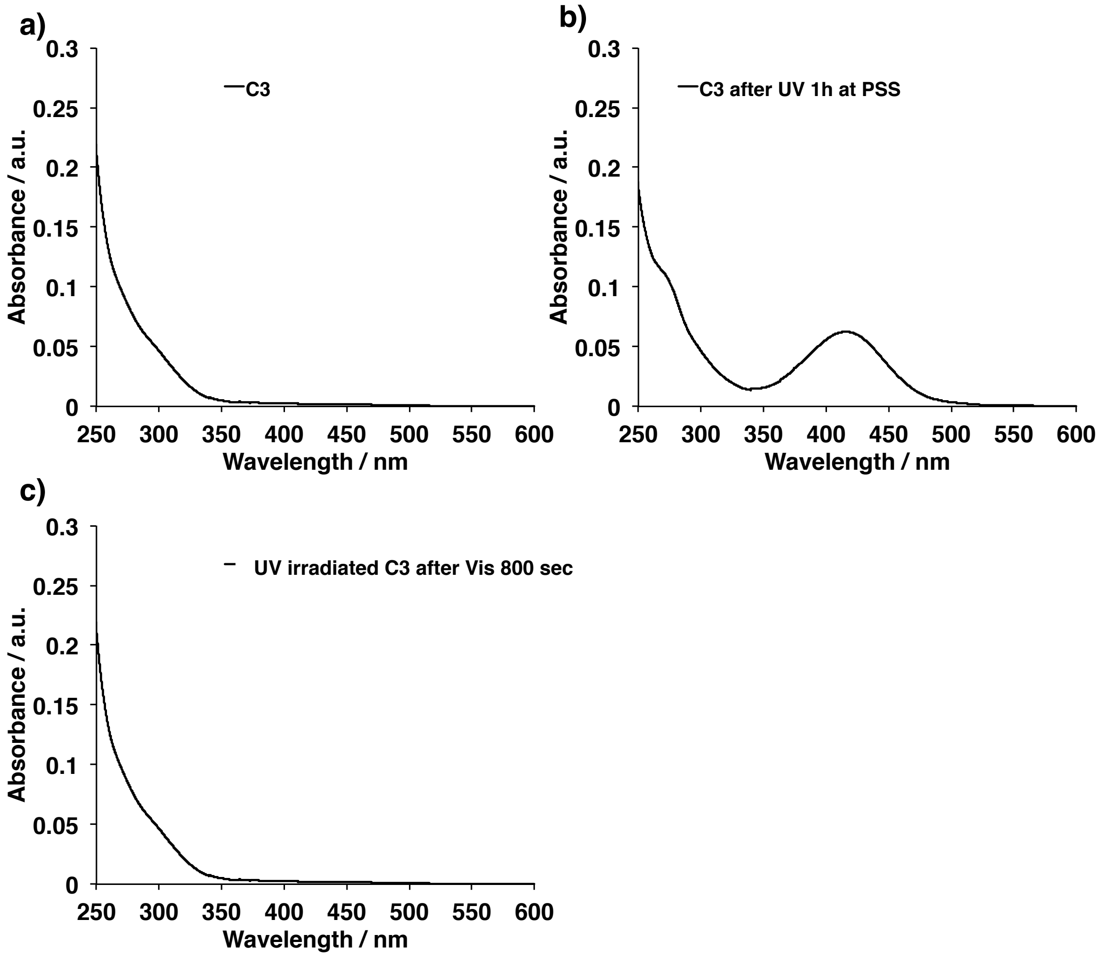

Supplementary Fig. 9. UV-Vis spectra of a) **C3**, b) after UV irradiation of a) for 1 h, and c) after irradiation of visible light for b) 800 sec.

Sonication Procedure

General procedure of ultrasonication experiments.

Polymer was dissolved in anhydrous acetonitrile (2 mg/mL), and it was then transferred to a dried glass tube, equipped with sonication probe. The glass tube was cooled to 0 ºC with ice bath, and sparged with Ar for 15 min prior to ultrasound irradiation. Pulsed sonication (1 s on, 1 s off) was carried out using 12% amplitude (10.6 $W \cdot cm^{-2}$). Aliquots (1.0 mL) were removed at 0, 7.5, 15, 22.5, 30, 37.5, and 45 min (sonication "on" time) and the solvent was removed under vacuum. Each sample was redissolved in a THF 0.5 mL and analyzed by GPC. Ultrasonic intensity was calibrated using the method described by Berkowski *et al.*[2]

For GPC Analyses

Aliquots of ultrasonicated polymer solutions were removed at regular time intervals and the solution was dried under reduced pressure. The samples were then redissolved in THF to afford a 4 mg/mL solution, filtered through a 0.45 μm PTFE syringe filter, and analyzed by SEC. The rate constants of polymer cleavage (*k*) for polymers (Fig. 5) were calculated from the slope of the least squares linear regression of the data according to the method of Kryger et al.[3] using Supplementary Equation 2, where M_t is the number average molecular weight (M_n) of the sonicated sample at time *t*, M_0 is the initial number average molecular weight of the polymer, and *k* is the rate constant of polymer cleavage adjusted for the molecular weight of the monomer unit, *M* ($k = k'/M$).

$$\frac{1}{M_t} - \frac{1}{M_0} = kt \qquad (2)$$

^{1}H NMR spectra of sonicated polymers

Post-sonication ^{1}H NMR spectrum of C1

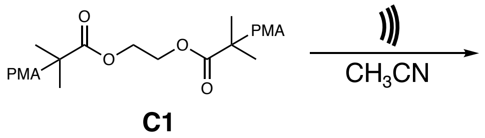

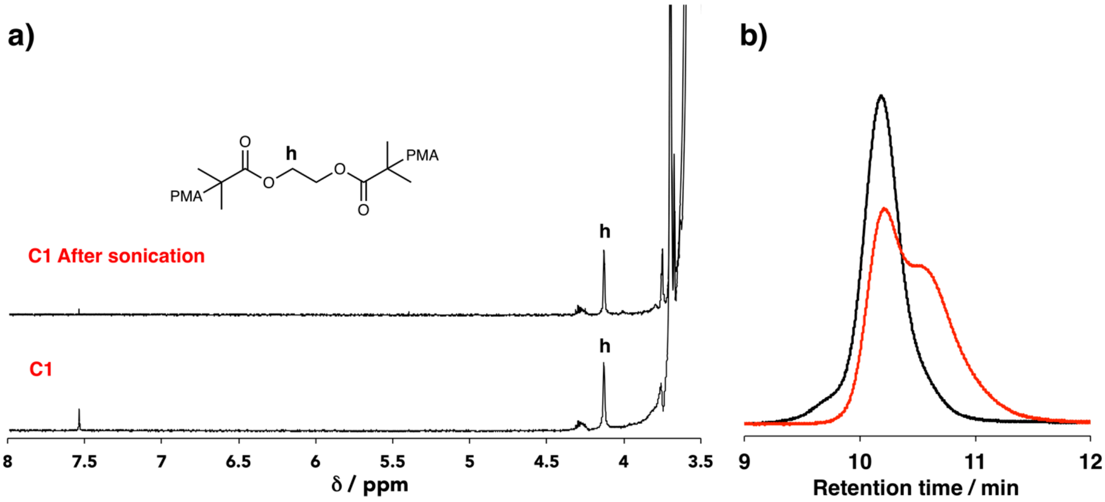

Supplementary Fig. 10. a) ^{1}H NMR spectra (500 MHz, CD_3CN, 25 ºC) of **C1** before and after 45 min of sonication. b) GPC curves of **C1** before (black) and after (red) 45 min of sonication.

Post-sonication ^{1}H NMR spectrum of C2

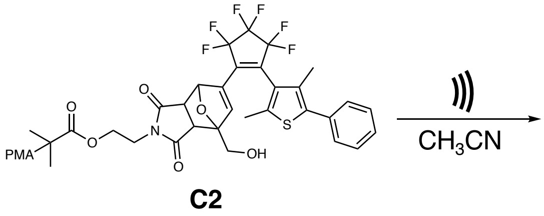

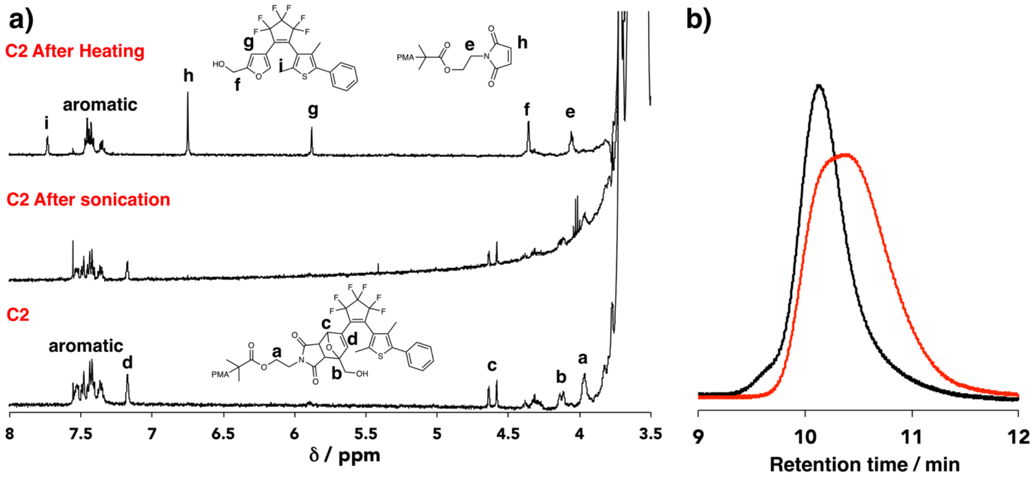

Supplementary Fig. 11. ^{1}H NMR spectra (500 MHz, CD_3CN, 25 ºC) of **C2** before and after 45 min of sonication. b) GPC curves of **C2** before (black) and after (red) 45 min of sonication.

Post-sonication ^{1}H NMR spectrum of C3

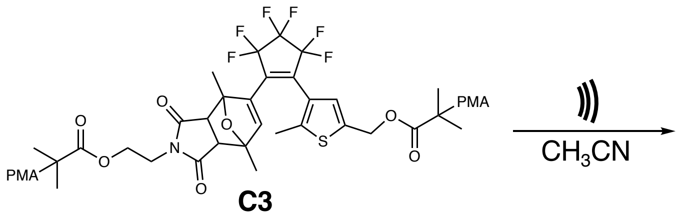

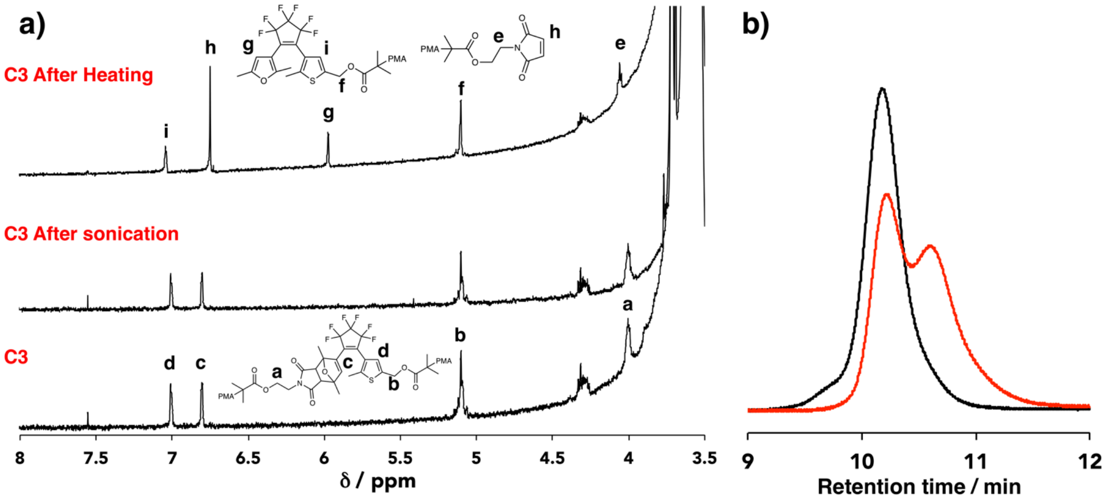

Supplementary Fig. 12. ^{1}H NMR spectra (500 MHz, CD_3CN, 25 ºC) of **C3** before and after 45 min of sonication. b) GPC curves of **C3** before (black) and after (red) 45 min of sonication.

UV-Vis spectra of sonicated polymers

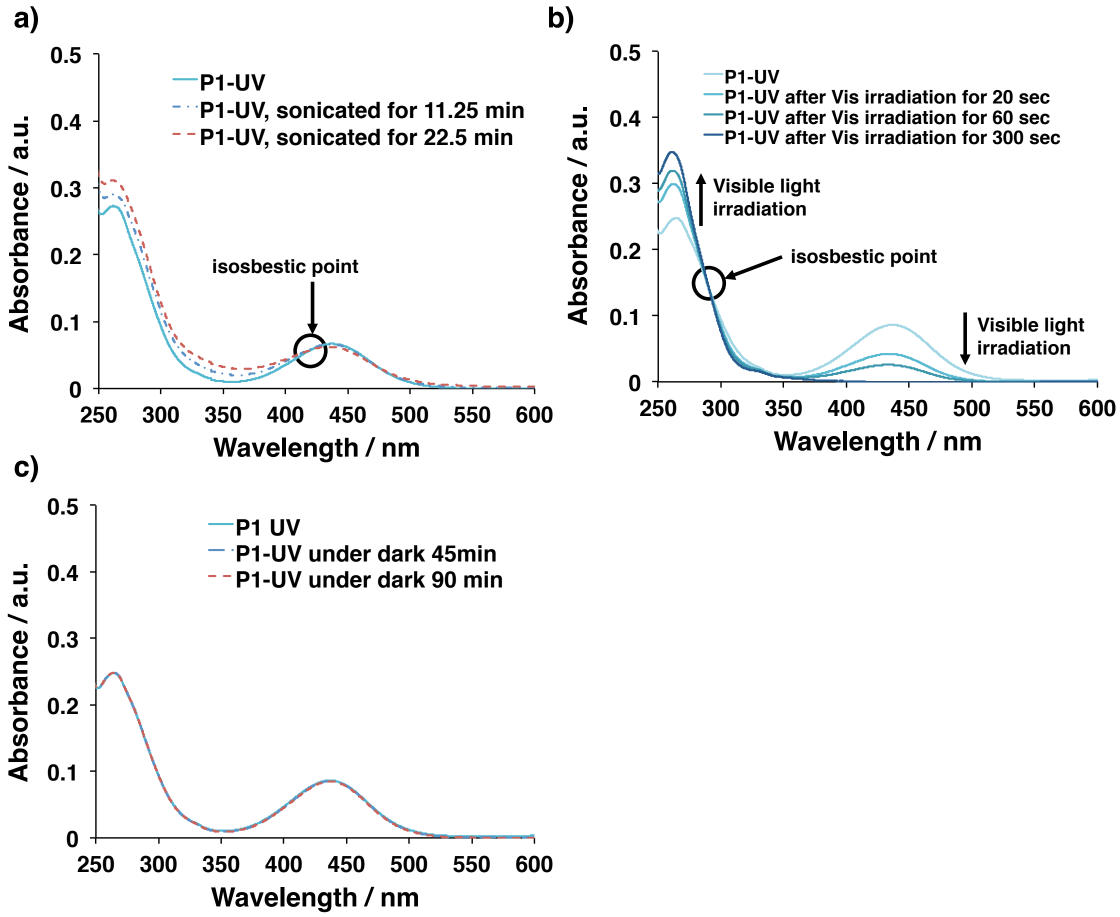

Supplementary Fig. 13. The change of UV-Vis spectra of UV irradiated **P1** (**P1-UV**) containing 60% of ring-closed DAE/DA (a) under sonication, (b) under visible light irradiation, and (c) under dark condition.

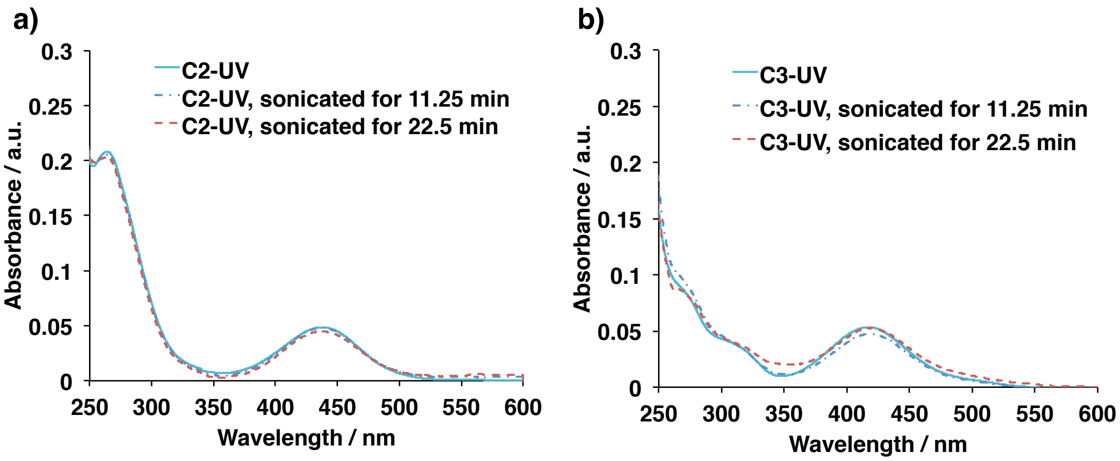

Supplementary Fig. 14. UV-Vis spectra of (a) UV irradiated **C2** (**C2-UV**) containing 42% of ring-closed DAE/DA and (b) UV irradiated **C3** (**C3-UV**) containing 53% of ring-closed DAE/DA under sonication.

CoGEF Analysis

CoGEF calculations were performed using Gaussian 16 according to previously reported methods.[3-5] Ground state energies were calculated using DFT at the UB3LYP 6-31G* level of theory. Starting from the equilibrium geometry of the unconstrained molecule (Energy = 0 kJ/mol), the distance between the methyl esters of the truncated structure was increased in increments of approximately 0.1 Å (0.05 Å in the area surrounding the breaking point) and the energy was minimized at each interval.

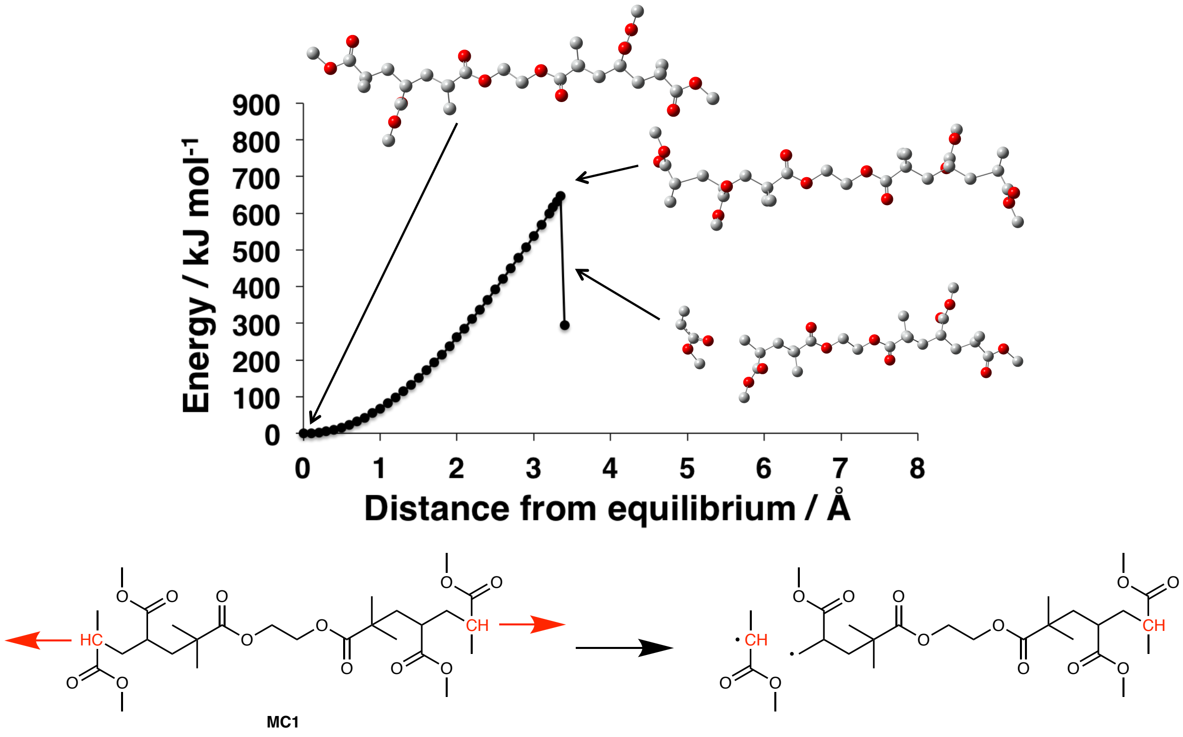

Supplementary Fig. 15. The top graph shows CoGEF analysis of model of **C1** (**MC1**) with the corresponding 3D structures at relax state, at E_{max}, and after bond cleavage (hydrogen atoms omitted for clarity). Solid line is a guide to the eyes. The lower scheme shows the 2D structure of **MC1** and its after cleavage.

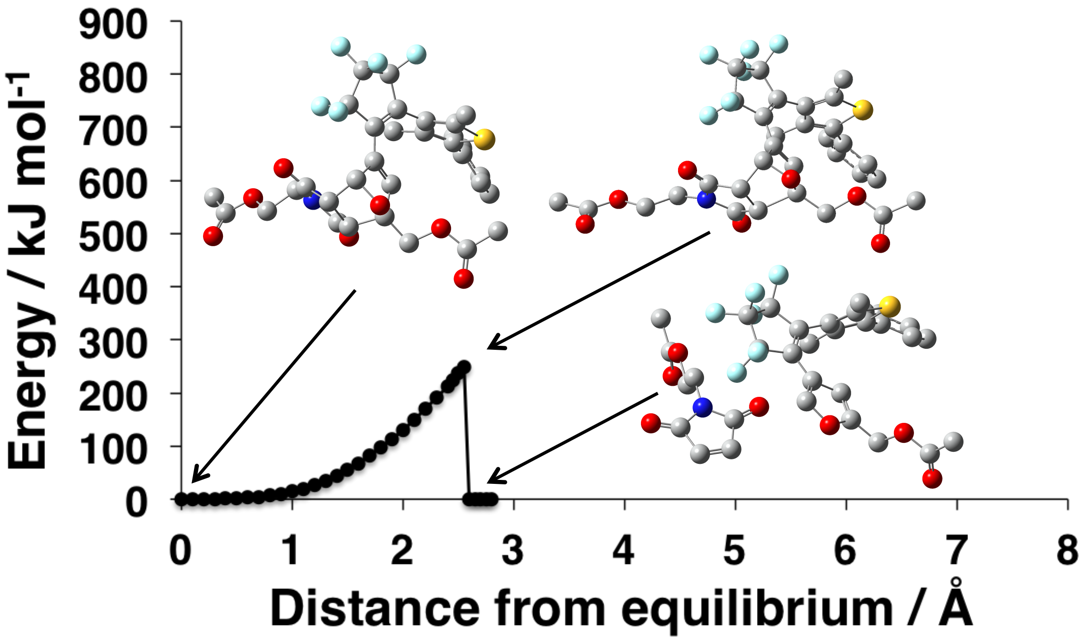

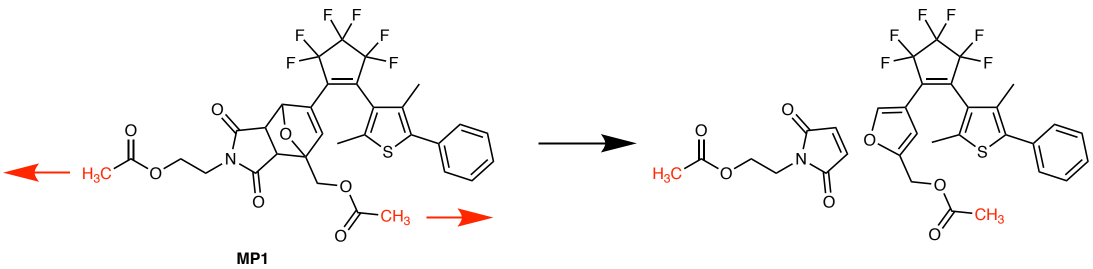

Supplementary Fig. 16. The top graph shows CoGEF analysis of model of **P1** (**MP1**) with the corresponding 3D structures at relax state, at E_{max}, and after bond cleavage (hydrogen atoms omitted for clarity). Solid line is a guide to the eyes. The lower scheme shows the 2D structure of **MP1** and its after cleavage.

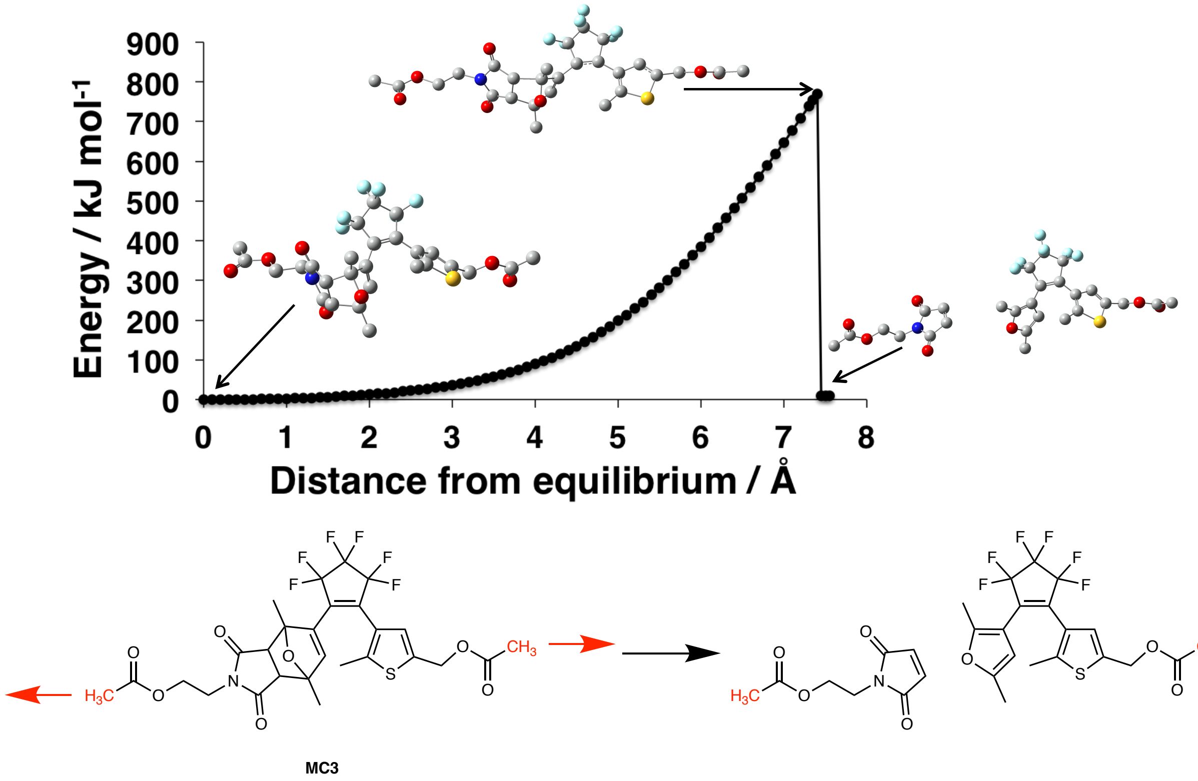

Supplementary Fig. 17. The top graph shows CoGEF analysis of model of **C3** (**MC3**) with the corresponding 3D structures at relax state, at E_{max}, and after bond cleavage (hydrogen atoms omitted for clarity). Solid line is a guide to the eyes. The lower scheme shows the 2D structure of **MC3** and its after cleavage.

Supplementary Table 2. E_{max} and F_{max} values for mechanophores studied herein

	E_{max} (kJ/mol)	F_{max} (nN)
**MP1**	248	3.97
**MC3**	770	5.20
**MC1**	647	5.15

(F_{max} values were determined using Beyer's method from the slope of the energy/elongation curve close to E_{max})[4]

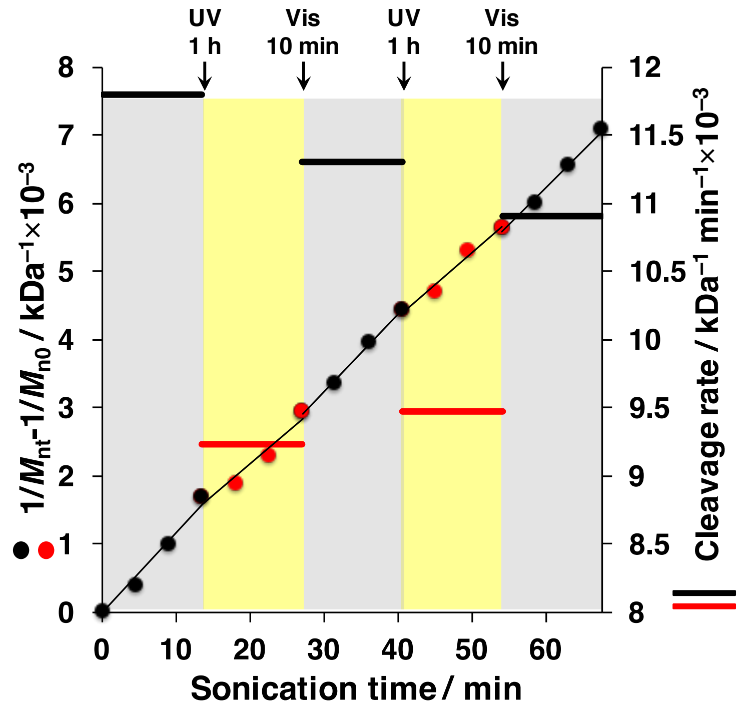

Supplementary Fig. 18. Photoregulation of the polymer chain scission upon exposure to pulsed ultrasound.

Synthesis of Initiators and their reactivity

Synthesis

2-(4-Bromofuran-2-yl)-1,3-dioxolane (1)[6]

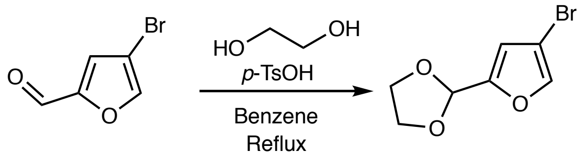

1

A benzene (100 mL) solution of 4-bromofuran-2-carbaldehyde (5.29 g, 30.3 mmol), ethylene glycol (8.43 mL, 151 mmol) and *p*-toluenesulfonic acid (10.0 mg, 0.0581 mmol) was refluxed with Dean–Stark apparatus for 5 h. The reaction mixture was washed twice with NaOH aq. (3.0 mol/L) and twice with water. Then, the organic layer was dried over anhydrous $MgSO_4$, filtered, and concentrated to provide the compound **1** as an orange liquid (5.79 g, 87%). ^1H NMR (300 MHz, $CDCl_3$) δ/ppm = 7.42 (s, 1H), 6.49 (s, 1H), 5,89 (s, 1H), 4.12-3.98 (m, 4H).

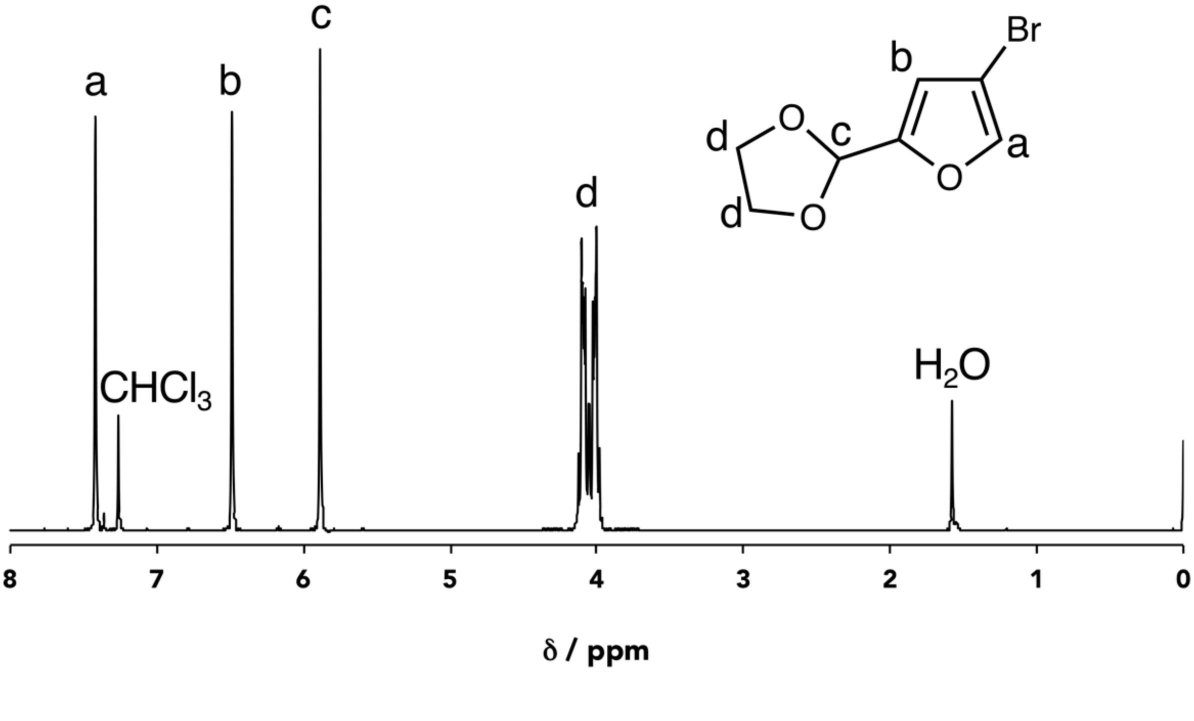

Supplementary Fig. 19. ^1H NMR spectrum of compound **1** ($CDCl_3$, 300 MHz, 25 ºC).

2,4-Dimethyl-3-(perfluorocyclopent-1-en-1-yl)-5-phenylthiophene (2)[7]

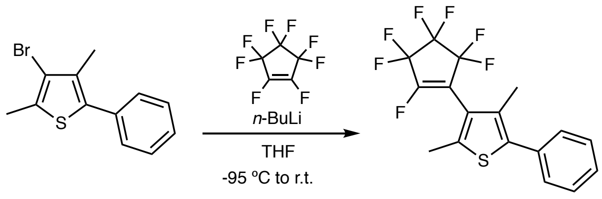

2

To a stirred anhydrous THF (50 mL) solution of 3-bromo-2,4-dimethyl-5-phenylthiophene (5.40 g, 20.2 mmol) was added dropwise a 1.60 M *n*-BuLi/hexane solution (13.8 mL, 22.2 mmol) at -95 ºC under nitrogen atmosphere. After stirring for 15 min, to an anhydrous THF (300 mL) solution of octafluorocyclopentene (10.7 g, 50.5 mmol) was added dropwise the above solution. The reaction mixture was stirred for 30 min, allowed to warm to room temperature with stirring, and quenched with water (20 mL) after 15 h. The mixture was concentrated, and extracted with ethyl acetate. Then, the organic layer was dried over anhydrous $MgSO_4$, filtered, and concentrated. The crude product was purified by column chromatography on neutral silica gel using hexane as the eluent to afford 5.30 g (69%) of compound **2** as white solid. ^{1}H NMR (300 MHz, $CDCl_3$) δ/ppm = 7.45-7.33 (m, 5H), 2,39 (s, 3H), 2.11 (s, 3H). ^{13}C NMR (75 MHz, $CDCl_3$) δ/ppm = 140.26, 136.88, 133.91, 132.13, 129.38, 128.79, 127.84, 120.96, 14.10, 13.89.

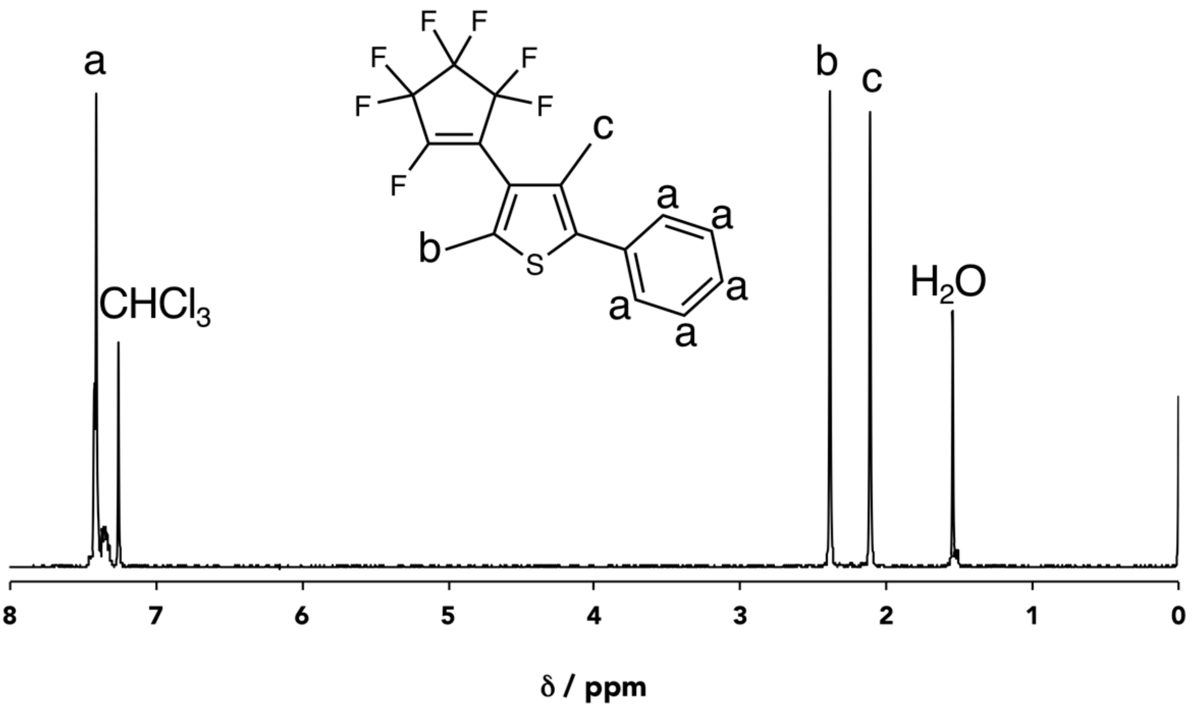

Supplementary Fig. 20. ^{1}H NMR spectrum of compound **2** ($CDCl_3$, 300 MHz, 25 ºC).

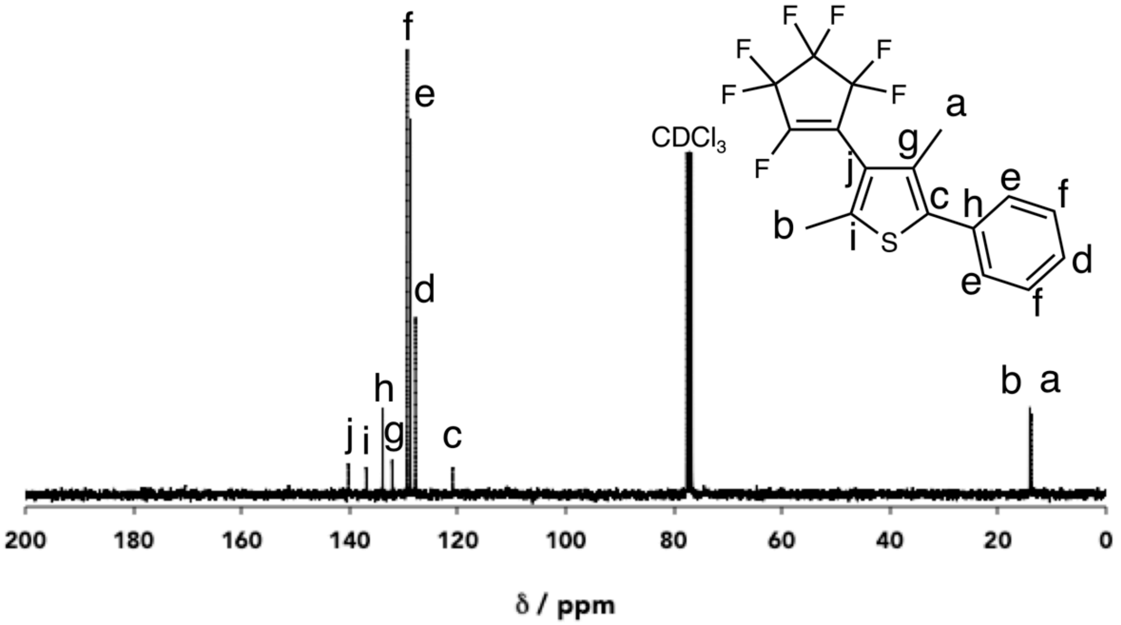

Supplementary Fig. 21. ^{13}C NMR spectrum of compound **2** ($CDCl_3$, 75 MHz, 25 ºC).

1-{4-[2-(1,3-Dioxolane)furyl]}-2-[3-(2,4-dimethyl-5-phenylthienyl)]hexafluorocyclopentene (3)

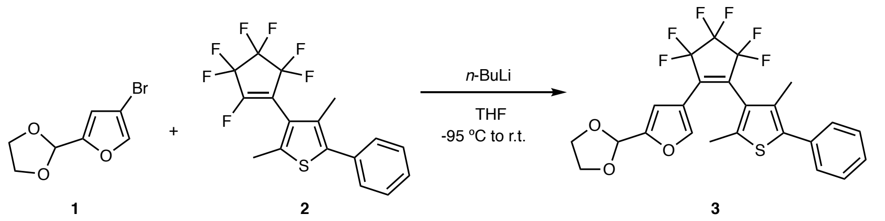

To a stirred anhydrous THF (200 mL) solution of compound **1** (3.04 g, 13.9 mmol) was added dropwise a 1.60 M *n*-BuLi/hexane solution (9.54 mL, 15.3 mmol) at -95 ºC under nitrogen atmosphere. After stirring for 15 min, to an anhydrous THF (250 mL) solution of compound **2** (5.28 g, 13.9 mmol) was added dropwise the above solution. The reaction mixture was stirred for 30 min, allowed to warm to room temperature with stirring, and quenched with water (20 mL) after 15 h. The mixture was concentrated, and extracted with ethyl acetate. Then, the organic layer was dried over anhydrous $MgSO_4$, filtered, and concentrated. The crude product was purified by column chromatography on neutral silica gel using hexane/ethyl acetate (v/v=19/1) as the eluent to afford 4.86 g (70%) of compound **3** as a crude mixture and used for subsequent reaction. ^1H NMR (300 MHz, $CDCl_3$) δ/ppm = 7.54 (s, 1H), 7.44-7.34 (m, 5H), 6.12 (s, 1H), 5.83 (s, 1H), 4.08-3.95 (m, 4H), 2.29 (s, 3H), 2.03 (s, 3H).

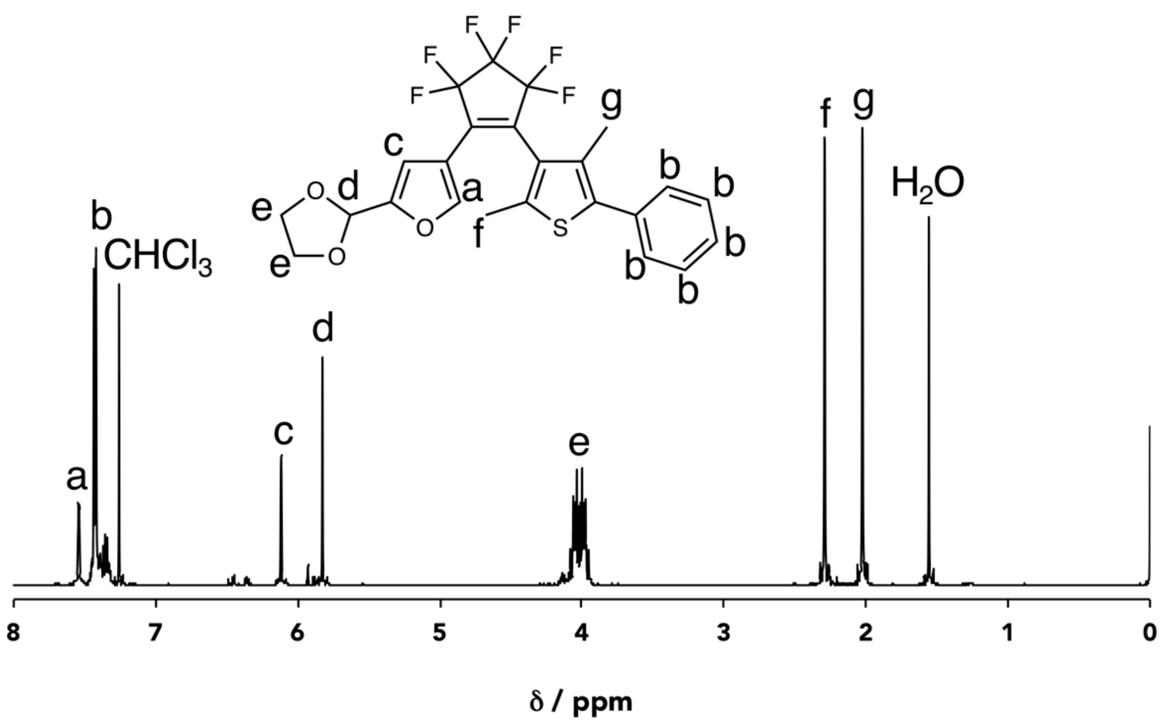

Supplementary Fig. 22. ^{1}H NMR spectrum of compound **3** ($CDCl_3$, 300 MHz, 25 ºC).

1-[4-(2-Hydroxymethlyfuryl)]-2-[3-(2,4-dimethyl-5-phenylthienyl)]hexafluorocyclopentene (4)

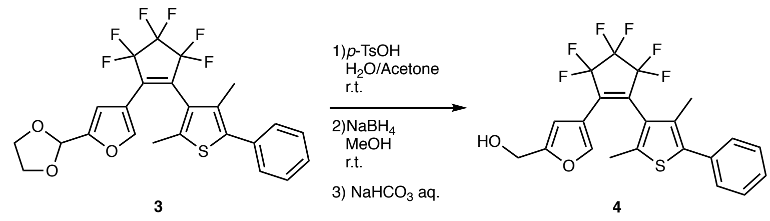

An acetone (20.0 mL) solution of compound **3** (1.62 g, 3.24 mmol), water (6.00 mL), and *p*-toluenesulfonic acid (10.0 mg, 0.0581 mmol) was stirred for 28 h at 40 ºC. The reaction was quenched with saturated aqueous sodium hydrogen carbonate. The reaction mixture was concentrated, and extracted with ethyl acetate. Then, the organic layer was dried over anhydrous $MgSO_4$, filtered, and concentrated. The obtained orange liquid was dissolved in methanol (30.0 mL), and the $NaBH_4$ (167 mg, 4.42 mmol) was added to the solution and the mixture was stirred for 5 min. at 0 ºC. The reaction was quenched with saturated aqueous sodium hydrogen carbonate, and the reaction mixture was concentrated. The product was extracted with ethyl acetate, and the organic layer was dried over anhydrous $MgSO_4$, filtered, and concentrated to provide the compound **4** as an orange liquid (1.16 g 78%). ^{1}H NMR (300 MHz, $CDCl_3$) δ/ppm = 7.59 (s, 1H), 7.44-7.34 (m, 5H), 5.96 (s, 1H), 4.55,4.53 (d, J = 5.93 Hz 2H), 2.30 (s, 3H), 2.04 (s, 3H), 1.73 (t, J = 6.22 Hz, 1H). ^{13}C NMR (75 MHz, $CDCl_3$) δ/ppm = 155.93, 144.73, 138.06, 137.17, 134.05, 131.85, 129.19, 128.78, 127.71, 126.96, 114.59, 106.02, 57.06, 13.83, 13.79. FT-IR (KBr. cm^{-1}): 3317, 3063, 2926, 2869, 1649, 1604, 1538, 1501, 1444, 1408, 1337, 1275, 1193, 1126, 1069, 1002, 970, 913, 873, 827, 795, 763, 700, 553, 496,

458. MALDI-TOF: 458.85 $[M]^+$, calculated for $C_{22}H_{16}F_6O_2S$ $[M]^+$: 458.08.

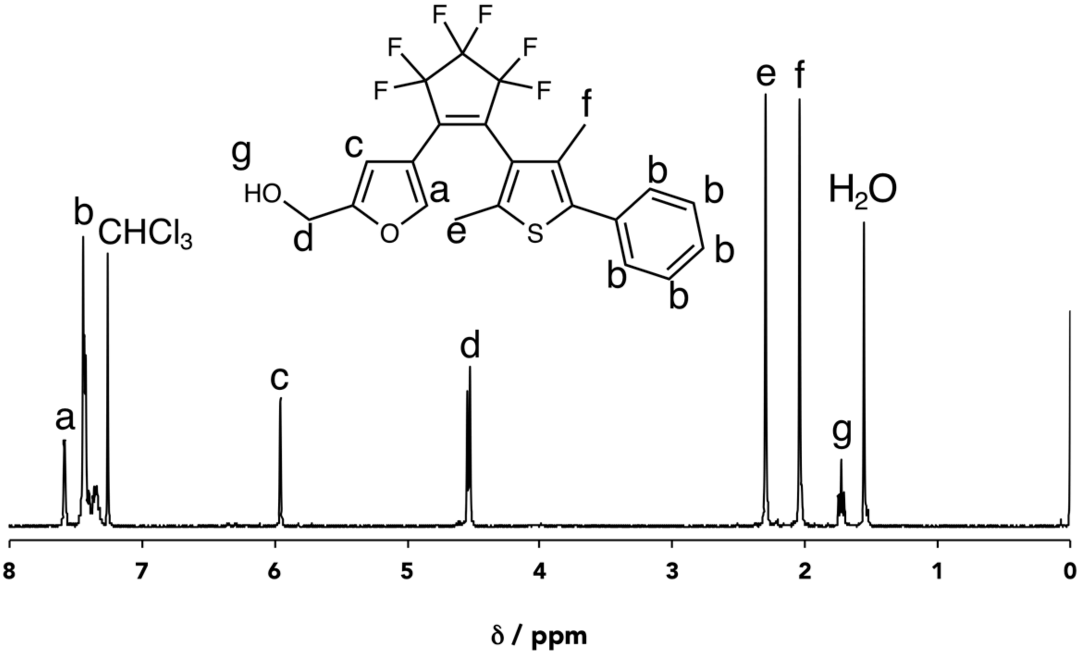

Supplementary Fig. 23. ^{1}H NMR spectrum of compound **4** ($CDCl_3$, 300 MHz, 25 ºC).

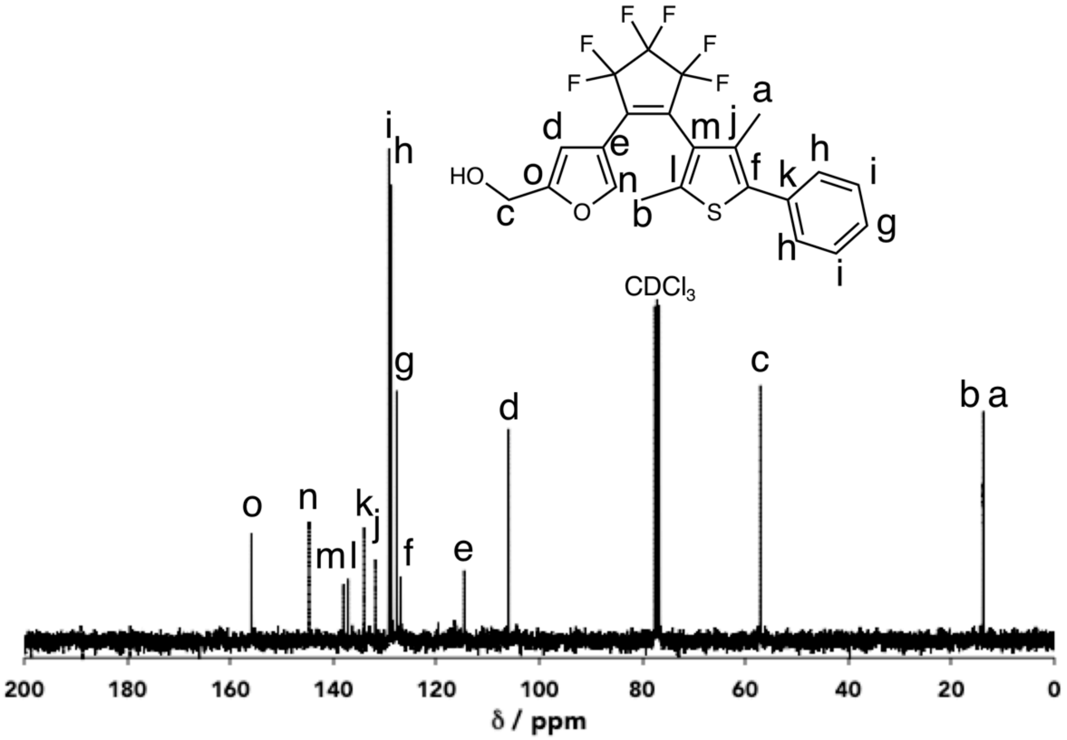

Supplementary Fig. 24. ^{13}C NMR spectrum of compound **4** ($CDCl_3$, 75 MHz, 25 ºC).

1-{8-(*N*-(2-Hydroxyethyl)-1-hydroxymethyl-7-oxatricyclo[5.2.1.0^{2,6}]deca-8-ene-3,5-dicarboimidyl)}-2-[3-(2,5-dimethyl-5-phenylthyenyl)]hexafluorocyclopentene ((DAE-open/DA)-diol)

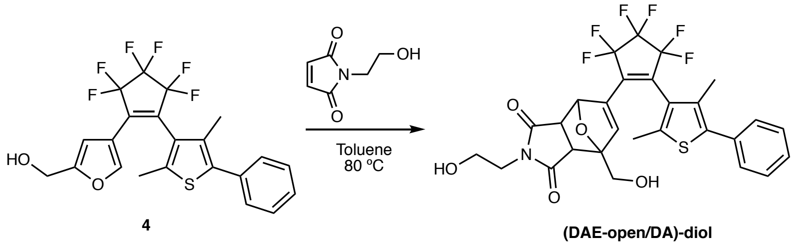

To a toluene (10.0 mL) solution of compound **4** (1.04 g, 2.27 mmol) was added *N*-(2-hydroxyethyl) maleimide (640 mg, 5.04 mmol), and the resulting solution was stirred at 80 ºC for 24 h. The reaction mixture was diluted with ethyl acetate and washed five times with water. The organic layer was dried over anhydrous $MgSO_4$, filtered, and concentrated. The crude product was purified by column chromatography on silica gel (hexane/ethyl acetate (v/v = 1/1 to 1/2)) to afford 1.29 g of DAE/DA-diol as yellow powder in 95% yield. ^{1}H NMR (500 MHz, $CDCl_3$) δ/ppm = 7.52-7.36 (m, 5H), 7.15 (s, 1H), 4.83,4.73 (d, J = 41.91 Hz, 1H), 4.11-4.02 (m, 2H), 3.74-3.65 (m, 4H), 3.07-3.02 (dd, J = 7.93, 6.56 Hz, 1H), 2.75-2.72 (dd, J = 6.56 Hz, 3.59 Hz, 1H), 2.75 (m, 1H), 2.37 (s, 3H), 2.09 (s, 3H), 1.90 (q, J = 5.8 Hz, 6.48 Hz, 1H). ^{13}C NMR (75 MHz, $CDCl_3$) δ/ppm = 175.48, 174.68, 143.68, 138.89, 138.57, 138.35, 133.75, 131.88, 131.14, 129.63, 129.47, 128.76, 128.71, 127.93, 125.57, 125.44, 92.28, 80.58, 80.49, 77.36, 60.06, 59.92, 49.40, 47.75, 41.82, 14.31, 13.85. FT-IR (KBr. cm^{-1}): 3458, 3087, 2949, 1776, 1704, 1604, 1436, 1400, 1338, 1270, 1191, 1126, 1059, 982, 868, 762, 702, 671, 580, 551, 502. MALDI-TOF: 622.46 [M+Na]$^+$, calculated for $C_{28}H_{23}F_6NO_5S$ [M+Na]$^+$: 622.11.

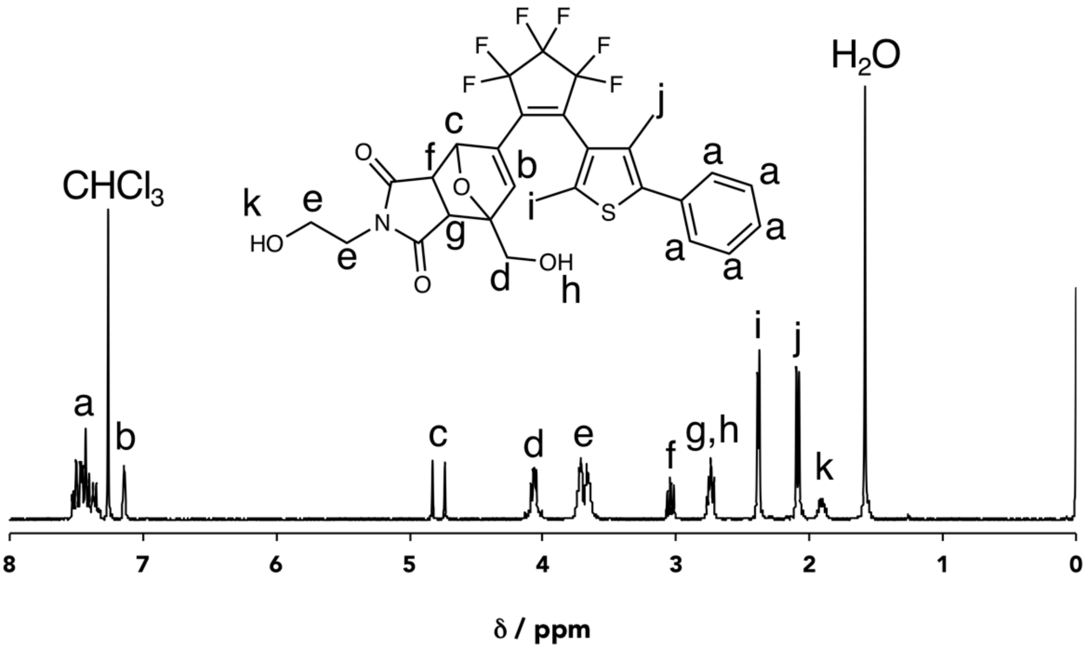

Supplementary Fig. 25. ^{1}H NMR spectrum of (DAE-open/DA)-diol ($CDCl_3$, 300 MHz, 25 ºC).

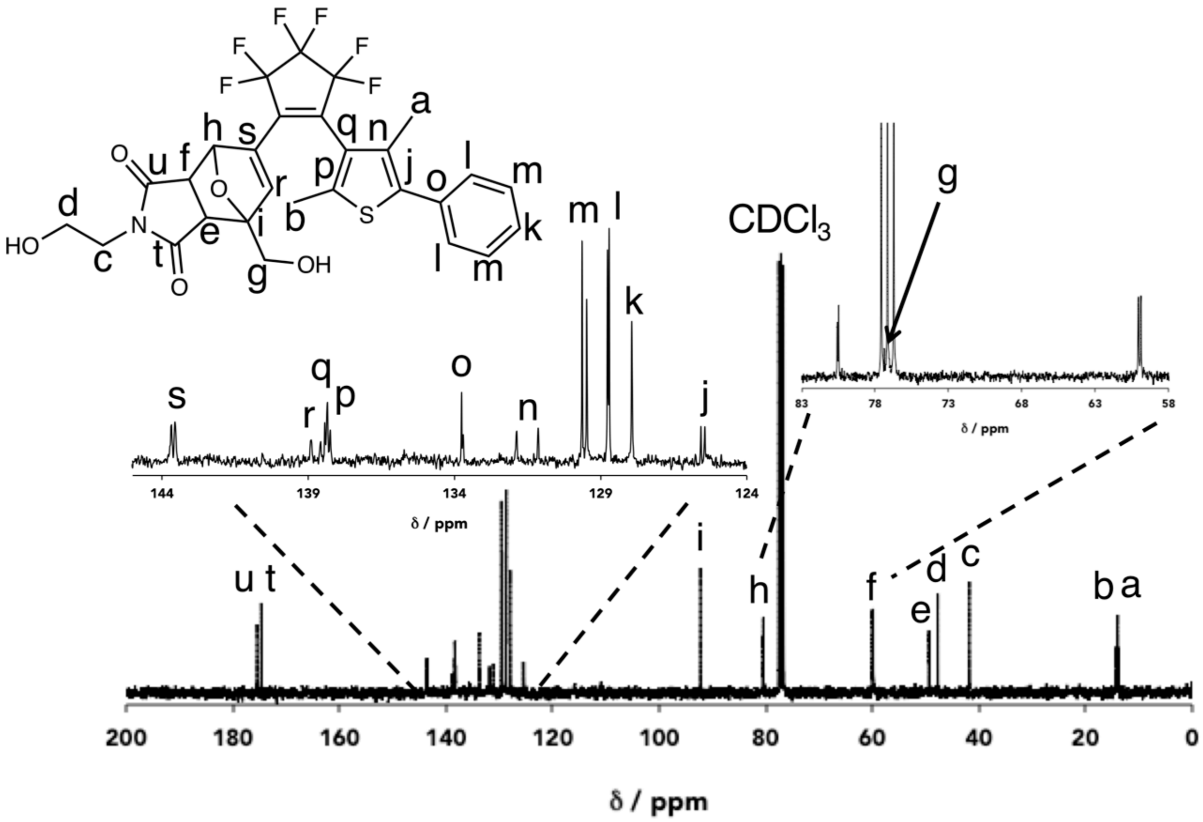

Supplementary Fig. 26. ^{13}C NMR spectrum of (DAE-open/DA)-diol ($CDCl_3$, 75 MHz, 25 ºC).

1-{8-(*N*-(2-(2-Bromoisobutyroxy)ethyl)-1-(2-bromoisobutyroxy)methyl-7-oxatricyclo[5.2.1.0^{2,6}]deca-8-en e-3,5-dicarboimidyl)}-2-[-3-(2,5-dimethyl-5-phenylthyenyl)]hexafluorocyclopentene (DAE/DA-dibromide)

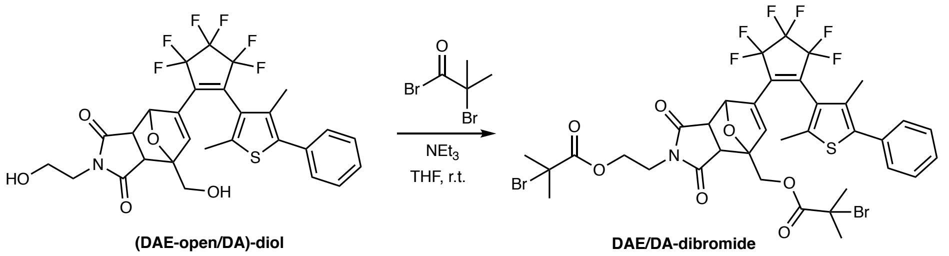

(DAE-open/DA)-diol DAE/DA-dibromide

DAE/DA-diol (140 mg, 234 μmol) was dissolved in anhydrous THF (4 mL) in round bottom flask. Triethylamine (289 μL, 2.01 mmol) was added via syringe followed by the dropwise addition of 2-bromoisobutyryl bromide (253 μL, 2.01 mmol). After stirring at room temperature for 5 h, the reaction mixture was filtered. The filtrate was concentrated under reduced pressure, diluted with dichloromethane and washed twice with water. 50 mL of NaOH aq. (1.0 mol/L) added to the solution and stirred for 1 h. Then, organic layer was washed with water, dried over anhydrous $MgSO_4$, and concentrated to provide DAE/DA-dibromide as a yellow powder (199 mg, 95%). H NMR (500 MHz, $CDCl_3$) δ/ppm = 7.53-7.36 (m, 5H), 6.96 (s, 1H), 4.97,4.93 (d, J = 12.81 Hz, 1H), 4.83,4.77 (d, J = 19.83 Hz, 1H), 4.59,4.54 (d, J = 12.81 Hz 1H), 4.39-4.22 (m, 2H), 3.83-3.71 (m, 2H), 3.01-2.97 (dd, J = 4.57 Hz, 2.03 Hz, 1H), 2.73-2.70 (dd, J = 4.99

Hz, 1.60 Hz, 1H), 2.36 (s, 3H), 2.08 (s, 3H), 1.92 (s, 6H), 1.81 (s, 6H). ^{13}C NMR (125 MHz, $CDCl_3$) δ/ppm = 173.51, 172.91, 171.53, 171.07, 142.01, 138.96, 138.8, 138.54, 138.39, 133.69, 131.74, 131.03, 129.63, 128.78, 128.74, 127.98, 125.37, 90.34, 80.69, 62.21, 55.72, 55.08, 49.42, 48, 38.06, 30.75, 30.73, 30.65, 30.57, 30.1, 14.13, 13.87. FT-IR (KBr. cm^{-1}): 2976, 2924, 1739, 1710, 1463, 1397, 1338, 1273, 1195, 1160, 1113, 1057, 984, 871, 760, 701, 496, 425. HRMS (FAB): 895.0250 $[M]^+$, calculated for $C_{36}H_{33}Br_2F_6NO_7S$ $[M]^+$: 895.0249.

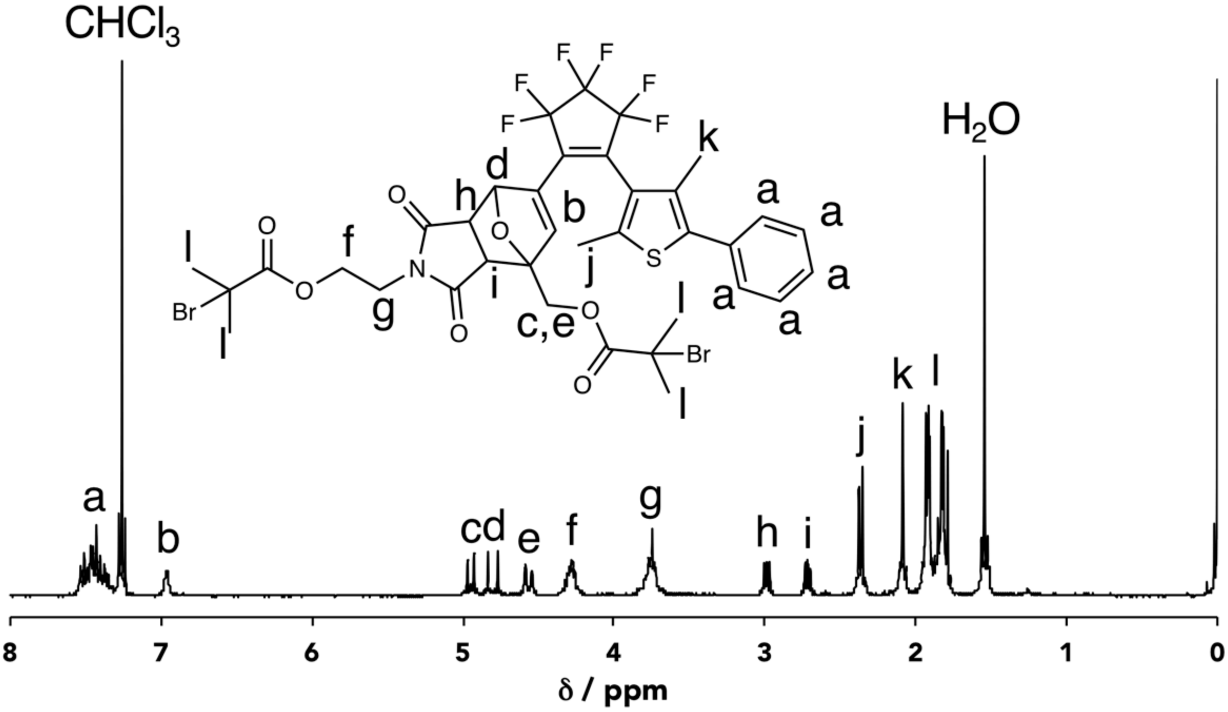

Supplementary Fig. 27. ^{1}H NMR spectrum of DAE/DA-dibromide ($CDCl_3$, 500 MHz, 25 ºC).

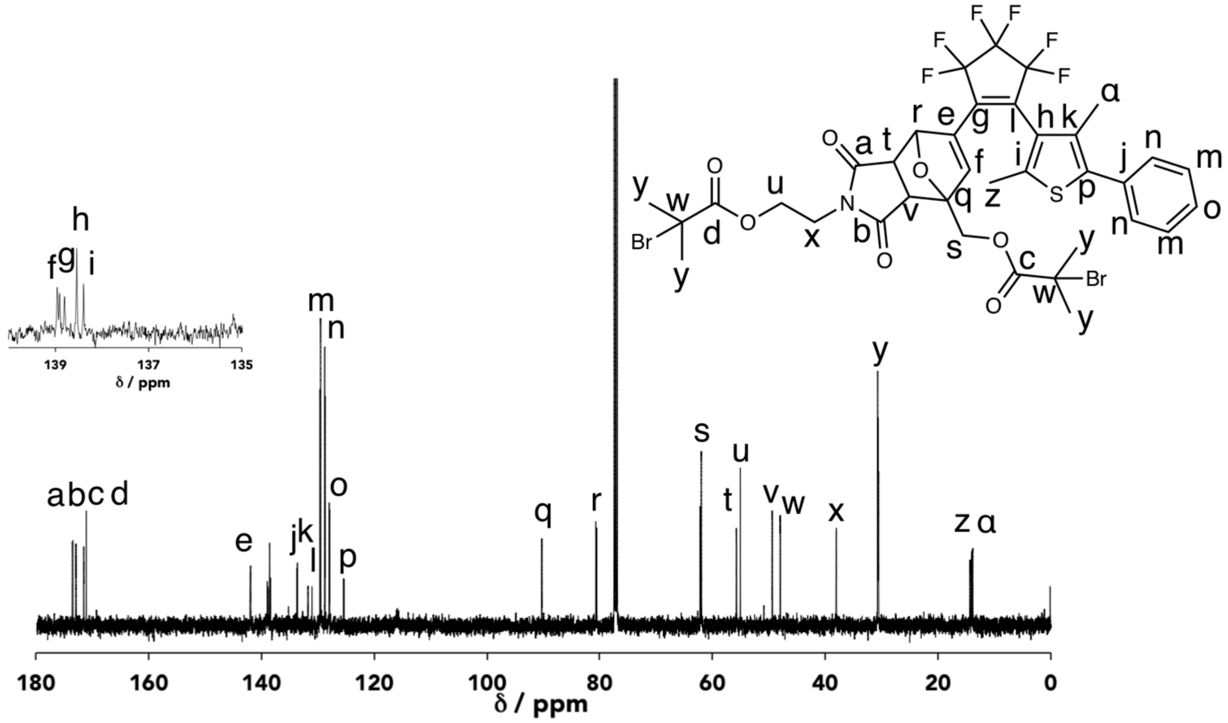

Supplementary Fig. 28. ^{13}C NMR spectrum of DAE/DA-dibromide ($CDCl_3$, 125 MHz, 25 ºC).

N-[2-(2-Bromoisobutyroxy)ethyl] maleimide (5)[8]

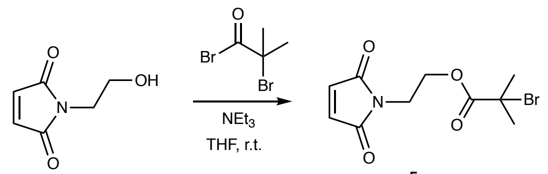

N-(2-hydroxyethyl) maleimide (120 mg, 847 μmol) was dissolved in anhydrous dichloromethane (2.5 mL) in round bottom flask. Triethylamine (119 μL, 847 μmol) was added via syringe followed by the dropwise addition of 2-bromoisobutyryl bromide (156 μL, 1.27 mmol). After stirring at room temperature for 1 h, the reaction mixture was filtered. The filtrate was concentrated under reduced pressure, diluted with dichloromethane and washed twice with water. 30 mL of NaOH aq. (1.0 mol/L) added to the solution and stirred for 1 h. Then, organic layer was washed with water, dried over anhydrous $MgSO_4$, and concentrated to provide compound **5** as a white solid (91.0 mg, 37%). ^1H NMR (500 MHz, $CDCl_3$) δ/ppm = 6.73 (s, 2H), 4.33 (t, J = 5.34 Hz, 2H), 3.86 (t, J = 5.42 Hz, 2H), 1.89 (s, 3H).

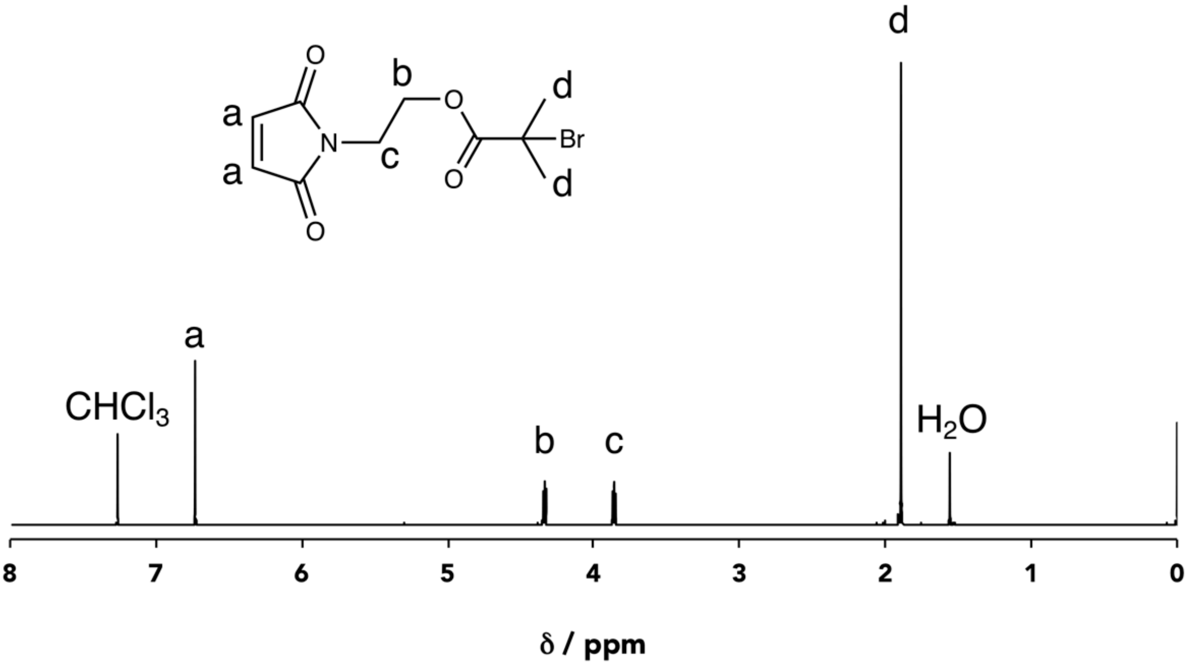

Supplementary Fig. 29. ^1H NMR spectrum of compound **5** ($CDCl_3$, 500 MHz, 25 ºC).

1-{8-(*N*-(2-(2-Bromoisobutyroxy)ethyl)-1-(2-hydroxymethyl)-7-oxatricyclo[5.2.1.0^{2,6}]deca-8-ene-3,5-dicarboimidyl)}-2-[-3-(2,5-dimethyl-5-phenylthyenyl)]hexafluorocyclopentene (6)

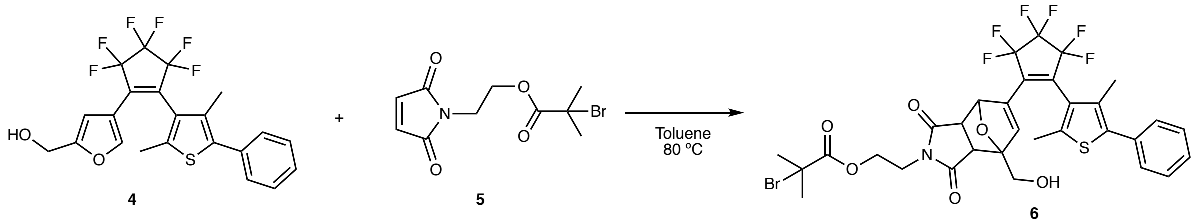

To a toluene (3.0 mL) solution of compound **4** (288 mg, 628 μmol) was added compound **5** (91.0 mg, 314 μmol), and the resulting solution was stirred at 80 ºC for 24 h. The crude product was purified by column chromatography on silica gel (hexane/ethyl acetate (*v*/*v* = 1/3)) to afford 211 mg of compound **6** as yellow powder in 90% yield. ^{1}H NMR (500 MHz, $CDCl_3$) *δ*/ppm = 7.54-7.34 (m, 5H), 7.15 (s, 1H), 7.78,4.70 (d, *J* = 43.49 Hz, 1H), 4.32-4.25 (m, 2H), 4.09-4.06 (m, 2H), 3.83-3.71 (m, 2H), 3.04-3.01(dd, *J* = 6.71 Hz, 1.83 Hz, 1H), 2.72-2.70 (dd, *J* = 5.04 Hz, 1.60 Hz, 1H), 2.71-2.67 (m, 1H), 2.37 (s, 3H), 2.09 (s, 3H), 1.81 (s, 6H). ^{13}C NMR (75 MHz, $CDCl_3$) *δ*/ppm = 174.75, 173.83, 171.53, 143.6, 138.81, 138.59, 138.5, 138.32, 133.7, 131.88, 131.12, 129.66, 128.78, 127.93, 125.52, 92.26, 92.23, 80.51, 80.34, 62.15, 60.27, 55.68, 49.4, 47.79, 38.08, 30.64, 14.34, 13.99. FT-IR (KBr. cm^{-1}): 3468, 2928, 1708, 1397, 1338, 1272, 1194, 1122, 1060, 983, 872, 765, 703, 481. HRMS (FAB): 747.0724 $[M]^+$, calculated for $C_{32}H_{28}BrF_6NO_6S$ $[M]^+$: 747.0725.

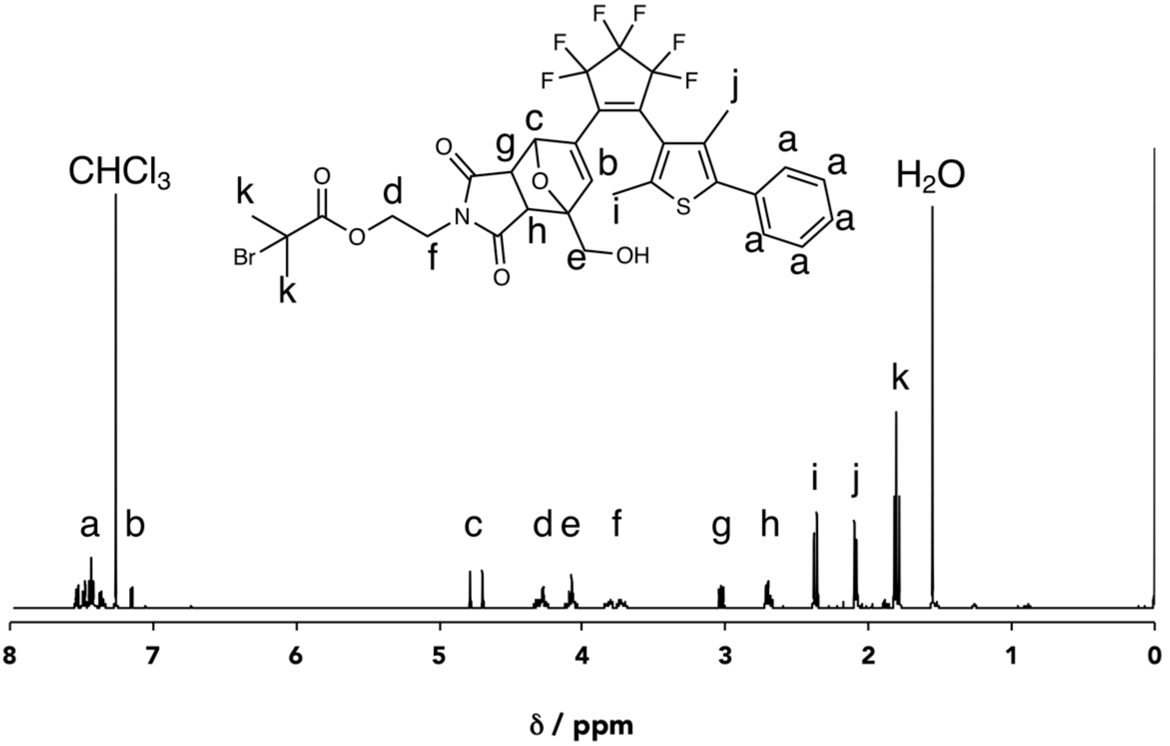

Supplementary Fig. 30. ^{1}H NMR spectrum of compound **6** ($CDCl_3$, 500 MHz, 25 ºC).

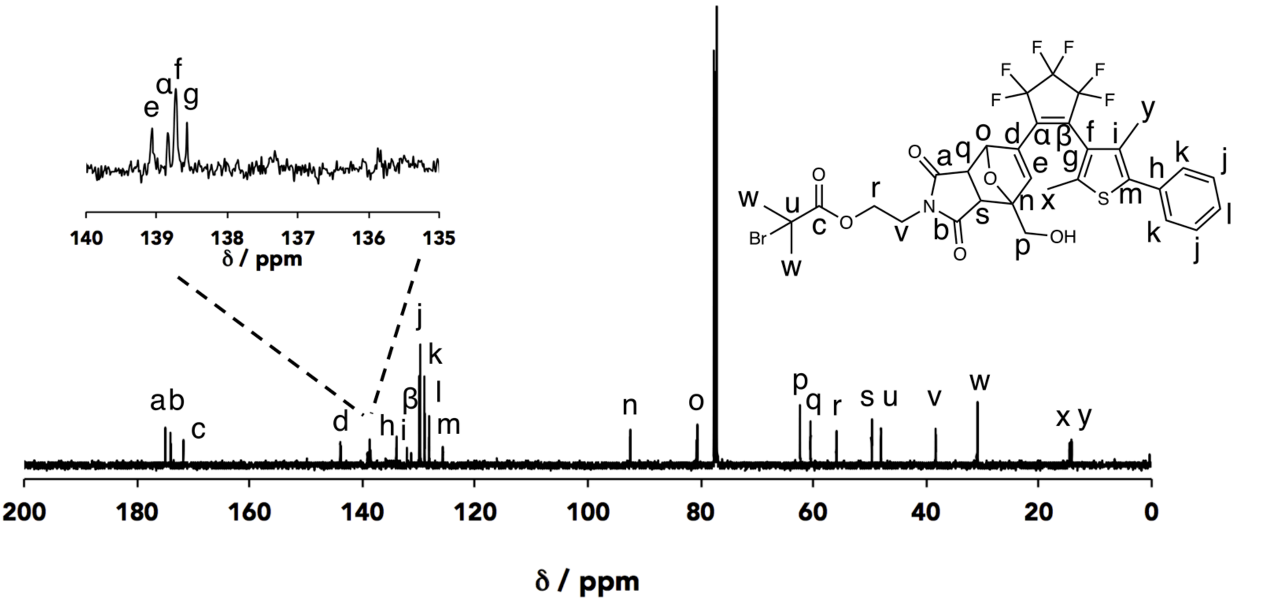

Supplementary Fig. 31. ^{13}C NMR spectrum of compound **6** ($CDCl_3$, 125 MHz, 25 ºC).

1-{8-(*N*-(2-(2-Bromoisobutyroxy)ethyl)-7-oxatricyclo[5.2.1.$0^{2,6}$]deca-8-ene-1,7-dimethyl-3,5-dicarboimidyl)}-2-[3-(2-methyl-5-(2-Bromoisobutyroxy)methylthyenyl)]hexafluorocyclopentene (7)

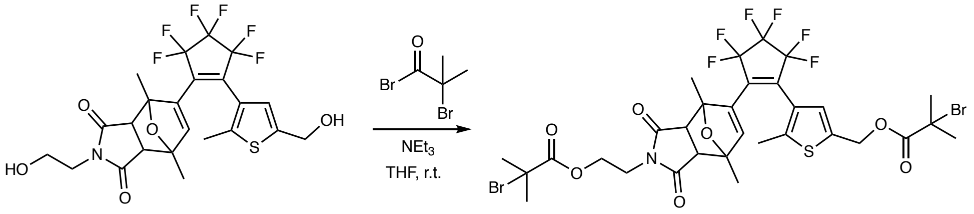

7

1-{8-(*N*-(2-Hydroxyethyl)-7-oxatricyclo[5.2.1.02,6]deca-8-ene-1,7-dimethyl-3,5-dicarboimidyl)}-2-[3-(2-methyl-5-hydroxymethylthyenyl)]hexafluorocyclopentene (DAE/DA-diol-2)[9] (230 mg, 428 μmol) was dissolved in anhydrous THF (15 mL) in round bottom flask. Triethylamine (132 μL, 942 μmol) was added via syringe followed by the dropwise addition of 2-bromoisobutyryl bromide (116 μL, 942 μmol). After stirring at room temperature for 1 h, the reaction mixture was filtered. The filtrate was concentrated under reduced pressure, diluted with dichloromethane and washed twice with water. 50 mL of NaOH aq. (1.0 mol/L) added to the solution and stirred for 1 h. Then, organic layer was washed with water, dried over anhydrous $MgSO_4$, and concentrated to provide compound **7** as a yellow solid (215 mg, 60%). ^1H NMR (500 MHz, $CDCl_3$) *δ*/ppm = 6.89 (s, 1H), 6.52 (s, 1H), 5.20 (s, 2H), 4.22 (t, *J* = 5.49 Hz, 2H), 3.68 (t, *J* = 5.49 Hz, 2H), 2.79,2.77 (d, *J* = 6.56 Hz, 1H), 2.48,2.46 (d, *J* = 6.96 Hz, 1H), 1.85 (s, 6H), 1.81 (s, 6H), 1.60 (s, 3H), 1.34 (s, 3H). ^{13}C NMR (75 MHz, $CDCl_3$) *δ*/ppm = 174.01, 173.83, 171.81, 171.59, 146.48, 143.24, 142.22, 136.80, 128.82, 124.34, 90.06, 87.85, 62.52, 61.84, 55.96, 55.78, 52.4, 52.37, 38.17, 31.04, 30.95, 15.96, 15.18, 14.99. FT-IR (KBr. cm^{-1}): 2981, 1775, 1739, 1708, 1443, 1395, 1337, 1275, 1196, 1158, 1110, 1041, 982, 876, 762, 703, 642, 483. HRMS (FAB): 833.0078 $[M]^+$, calculated for $C_{31}H_{31}Br_2F_6NO_7S$ $[M]^+$: 833.0092.

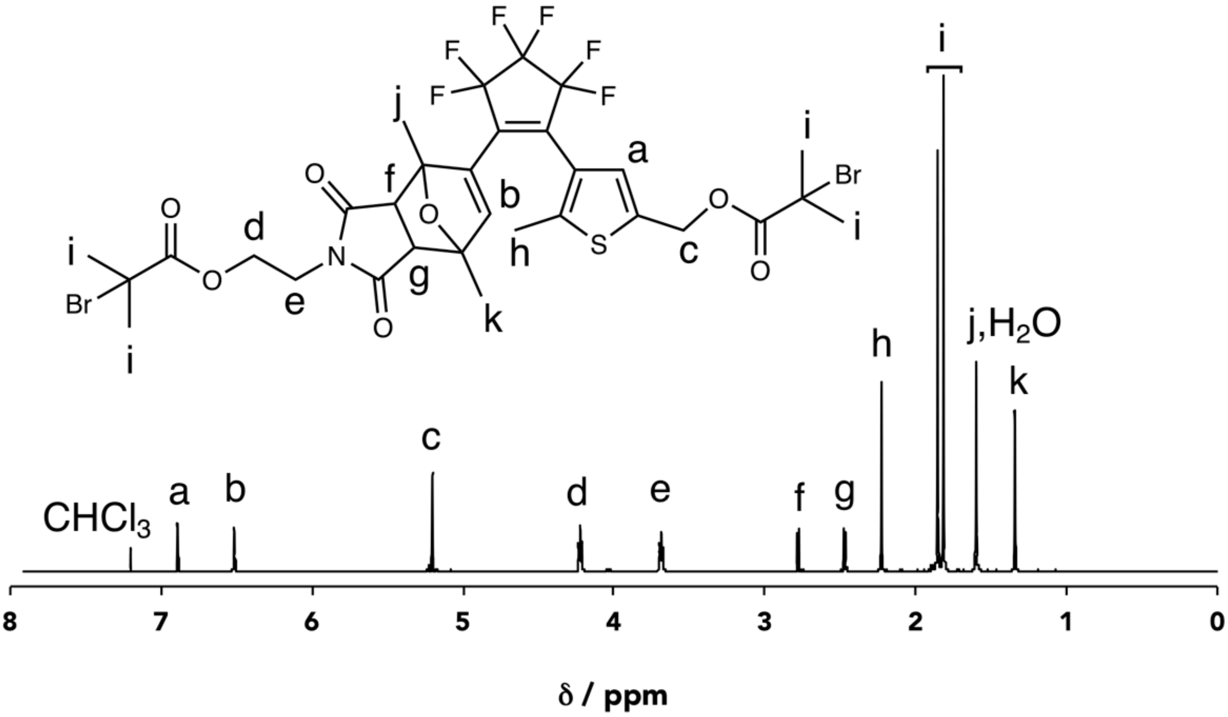

Supplementary Fig. 32. ^{1}H NMR spectrum of compound **7** ($CDCl_3$, 500 MHz, 25 ºC).

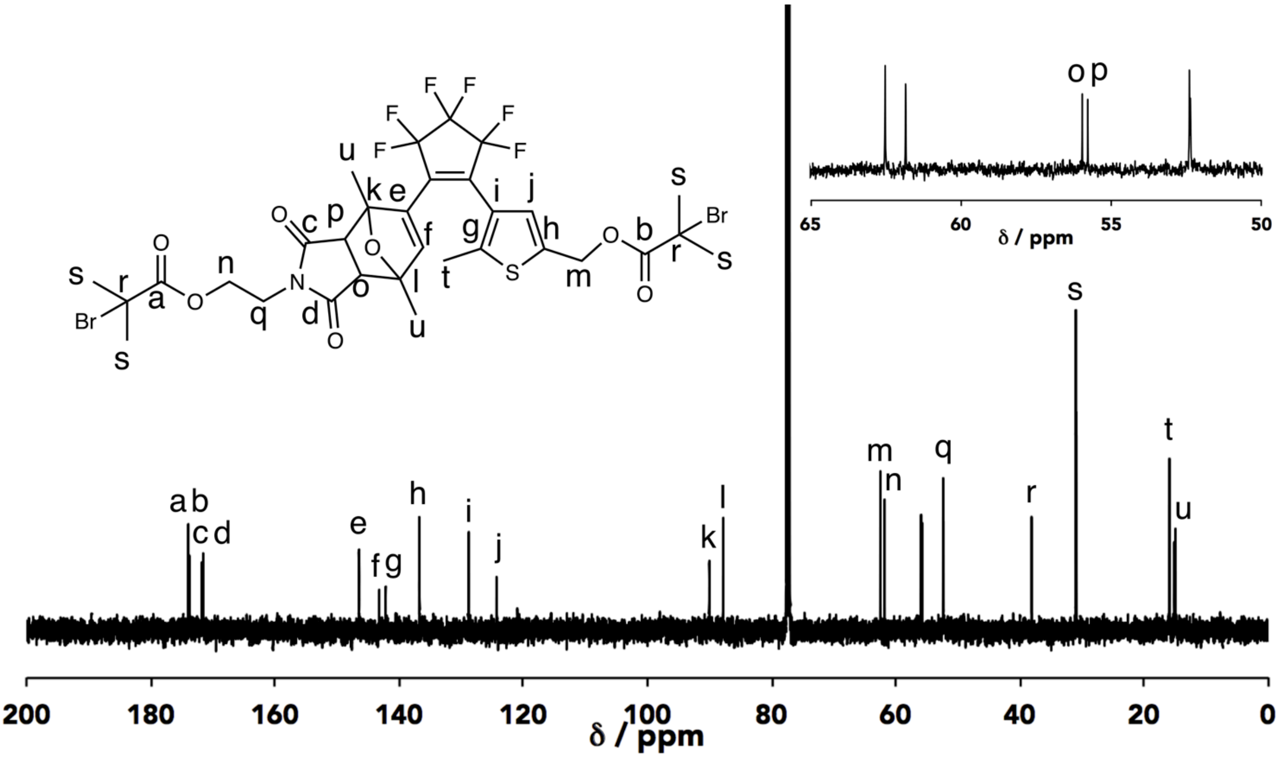

Supplementary Fig. 33. ^{13}C NMR spectrum of compound **7** ($CDCl_3$, 125 MHz, 25 ºC).

Supplementary References

1. Hickenboth, C. R. *et al.* Biasing reaction pathways with mechanical force. *Nature* **446,** 423–427 (2007).
2. Berkowski, K. L., Potisek, S. L., Hickenboth, C. R. & Moore, J. S. Ultrasound-Induced Site-Specific Cleavage of Azo-Functionalized Poly(ethylene glycol). *Macromolecules* **38,** 8975–8978 (2005).
3. Kryger, M. J., Munaretto, A. M. & Moore, J. S. Structure–Mechanochemical Activity Relationships for Cyclobutane Mechanophores. *J. Am. Chem. Soc.* **133,** 18992–18998 (2011).
4. Beyer, M. K. The mechanical strength of a covalent bond calculated by density functional theory. *J. Chem. Phys.* **112,** 7307–7312 (2000).
5. Davis, D. A. *et al.* Force-induced activation of covalent bonds in mechanoresponsive polymeric materials. *Nature* **459,** 68–72 (2009).
6. Ilovich, O. & Deutsch, J. A Selective Synthesis of 4-Bromo-2-furancarboxaldehyde and its Pinacolborane Derivative. *J. Heterocyclic Chem.* **42,** 1409–1411 (2005).
7. Shiozawa, T., Hossain, M. K., Ubukata, T. & Yokoyama, Y. Ultimate diastereoselectivity in the ring closure of photochromic diarylethene possessing facial chirality. *Chem. Commun.* **46,** 4785–4787 (2010).
8. Lucon, J., Edwards, E., Qazi, S., Uchida, M. & Douglas, T. Atom transfer radical polymerization on the interior of the P22 capsid and incorporation of photocatalytic monomer crosslinks. *Eur. Polym. J.* **49,** 2976–2985 (2013).
9. Kida, J., Imato, K., Goseki, R., Morimoto, M. & Otsuka, H. Photoregulation of Retro-Diels–Alder Reaction at the Center of Polymer Chains. *Chem. Lett.* **46,** 992–994 (2017).
